# Novel Interface Designs for Patient Monitoring Applications in Critical Care Medicine: Human Factors Review

**DOI:** 10.2196/15052

**Published:** 2020-07-03

**Authors:** Evismar Andrade, Leo Quinlan, Richard Harte, Dara Byrne, Enda Fallon, Martina Kelly, Siobhan Casey, Frank Kirrane, Paul O'Connor, Denis O'Hora, Michael Scully, John Laffey, Patrick Pladys, Alain Beuchée, Gearóid ÓLaighin

**Affiliations:** 1 Electrical & Electronic Engineering School of Engineering National University of Ireland, Galway Galway Ireland; 2 Human Movement Laboratory CÚRAM Centre for Research in Medical Devices National University of Ireland, Galway Galway Ireland; 3 Physiology, School of Medicine National University of Ireland, Galway Galway Ireland; 4 General Practice School of Medicine National University of Ireland, Galway Galway Ireland; 5 Irish Centre for Applied Patient Safety and Simulation University Hospital Galway Galway Ireland; 6 Mechanical Engineering School of Engineering National University of Ireland, Galway Galway Ireland; 7 Intensive Care Unit University Hospital Galway Galway Ireland; 8 University Hospital Galway Galway Ireland; 9 School of Psychology National University of Ireland, Galway Galway Ireland; 10 Anaesthesia School of Medicine National University of Ireland, Galway Galway Ireland; 11 Department of Anaesthesia & Intensive Care Medicine National University of Ireland, Galway Galway Ireland; 12 Centre Hospitalier Universitaire de Rennes (CHU Rennes) Rennes France; 13 Faculté de Médicine de l’Université de Rennes Rennes France

**Keywords:** interface design, usability, situation awareness, graphical display, satisfaction, response time, accuracy, anesthesiology, critical care, performance, ecological display

## Abstract

**Background:**

The patient monitor (PM) is one of the most commonly used medical devices in hospitals worldwide. PMs are used to monitor patients’ vital signs in a wide variety of patient care settings, especially in critical care settings, such as intensive care units. An interesting observation is that the design of PMs has not significantly changed over the past 2 decades, with the layout and structure of PMs more or less unchanged, with incremental changes in design being made rather than transformational changes. Thus, we believe it well-timed to review the design of novel PM interfaces, with particular reference to usability and human factors.

**Objective:**

This paper aims to review innovations in PM design proposed by researchers and explore how clinicians responded to these design changes.

**Methods:**

A literature search of relevant databases, following the Preferred Reporting Items for Systematic Reviews and Meta-Analyses guidelines, identified 16 related studies. A detailed description of the interface design and an analysis of each novel PM were carried out, including a detailed analysis of the structure of the different user interfaces, to inform future PM design. The test methodologies used to evaluate the different designs are also presented.

**Results:**

Most of the studies included in this review identified some level of improvement in the clinician’s performance when using a novel display in comparison with the traditional PM. For instance, from the 16 reviewed studies, 12 studies identified an improvement in the detection and response times, and 10 studies identified an improvement in the accuracy or treatment efficiency. This indicates that novel displays have the potential to improve the clinical performance of nurses and doctors. However, the outcomes of some of these studies are weakened because of methodological deficiencies. These deficiencies are discussed in detail in this study.

**Conclusions:**

More careful study design is warranted to investigate the user experience and usability of future novel PMs for real time vital sign monitoring, to establish whether or not they could be used successfully in critical care. A series of recommendations on how future novel PM designs and evaluations can be enhanced are provided.

## Introduction

**Figure 1:**
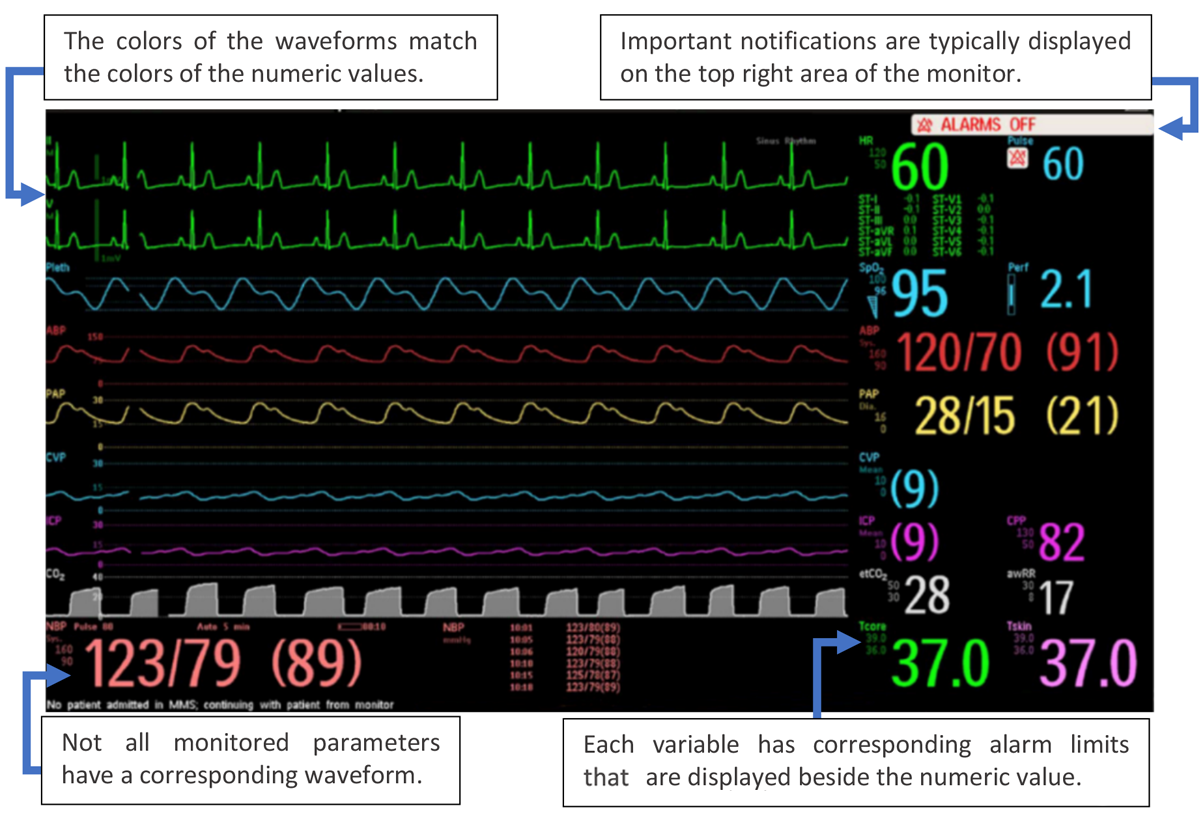
Example of a commercial patient monitor interface (Philips IntelliVue MX series). Each vital sign is color-coded (waveforms and numerical values). Depending on the make and model, additional information might also be displayed alongside the numerical values (eg, configured alarm limits and previous values for noninvasive blood pressure as seen in the image). The image was added with the permission of Philips. ABP: arterial blood pressure; awRR: airway respiratory rate; CPP: cerebral perfusion pressure; CVP: central venous pressure; etCO_2_: end-tidal carbon dioxide; HR: heart rate; ICP: intracranial pressure; NBP: noninvasive blood pressure; PAP: pulmonary artery pressure; SpO_2_: blood oxygen saturation; Tcore: core temperature; Tskin: skin temperature.

The patient monitor (PM) is one of the most commonly used medical devices in hospitals. It is used to monitor patients’ vital signs in a wide range of patient care environments. A typical PM interface is composed of two main elements: the waveform and the numerical values of the monitored parameters (Figure 1). The waveform element displays the analog signals for each parameter for a few seconds in a line graph. The numerical values element, on the other hand, represents the calculated value for each parameter in a numeric format and these values are continuously updated every few seconds or milliseconds, depending on the parameter. However, not all monitored parameters are displayed in both waveform and numeric form. For instance, noninvasive blood pressure (NIBP) is not continuously measured; hence, only the numerical value is presented, and this reading is updated every time this vital sign is measured according to clinical requirements.

The context in which PMs are used includes any clinical environment in which clinical caregivers provide critical care to patients. Such environments include the intensive care unit (ICU), emergency department, operating room (OR), cardiology unit, and during the transportation of a patient. Within these contexts of use, regular assessment of vital signs is crucial to identify patients at risk of serious adverse events as early as possible. During an anesthesia procedure, for example, the anesthesiologist needs to be able to quickly identify the changes in vital signs, whereas, in the ICU, if any of the vital signs become abnormal, nurses need to be immediately warned. In both cases, any delay in providing appropriate care or in making a clinical decision might result in severe consequences for the patient.

In such contexts of use, it is not uncommon for the primary users of a PM (nurses and doctors) to be under extreme pressure in terms of time, cognitive workload, and stress [1,2]. Correct decisions related to patient care based on information provided by the PM may need to be made in a short time. Coupled with this is the prevalence of work-related fatigue in these environments, which may increase the risk of use error when interacting with the PM [3]. For this reason, novel PMs need to reach the highest standards in usability and human factors, thereby facilitating enhanced user interaction and preventing potential risks related to use error. Good usability in medical device design is essential in avoiding potential risks associated with use error, as evidenced by the publication of standards documents such as IEC 62366-1/2, ANSI/AAMI HE75 and ISO 9241-210 210 [4-6]. HE75 makes frequent reference to the importance of usability engineering in the design of PMs. Usability is defined in ISO 9241-210 (section 2.13) as the “extent to which a system, product or service can be used by specified users to achieve specified goals with effectiveness, efficiency and satisfaction in a specified context of use” [6]. The study of human factors (section 2.5) is defined as “the scientific discipline concerned with the understanding of interactions among human and other elements of a system, and the profession that applies theory, principles, data and methods to design to optimize human well-being and overall system performance” [6].

**Figure 2:**
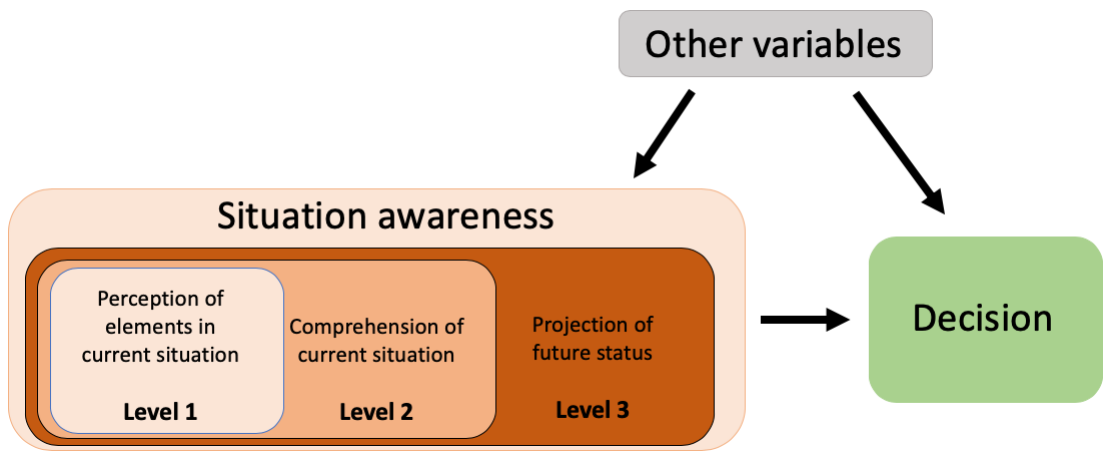
Part of the situation awareness model in dynamic decision making presented by Endsley (1995). This reflects how situation awareness influences decision making.

Given the importance of the decisions made in the critical care environment in response to displayed vital signs, it is imperative that PMs display the required information in a user-friendly manner to enable clinicians to fully comprehend the patient’s status. This level of comprehension will be referred to in this work as situation awareness (SA). According to Endsley [7], “Situation awareness is the perception of the elements in the environment within a volume of time and space, the comprehension of their meaning, and the projection of their status in the near future.” The concept of SA applies to many mission-critical tasks in various fields (eg, aviation, nuclear power plants, military combat systems, etc). In the context of using PMs in critical care medicine, SA level 1 (perception) is associated with the ability of the user to perceive the changes in vital signs; SA level 2 (comprehension) is associated with the ability of the user to understand the patient’s state based on the vital signs; and SA level 3 (projection) is associated with the ability of the user to predict the patient’s future state based on the current state. The flow of the SA process is illustrated in Figure 2.

**Figure 3:**
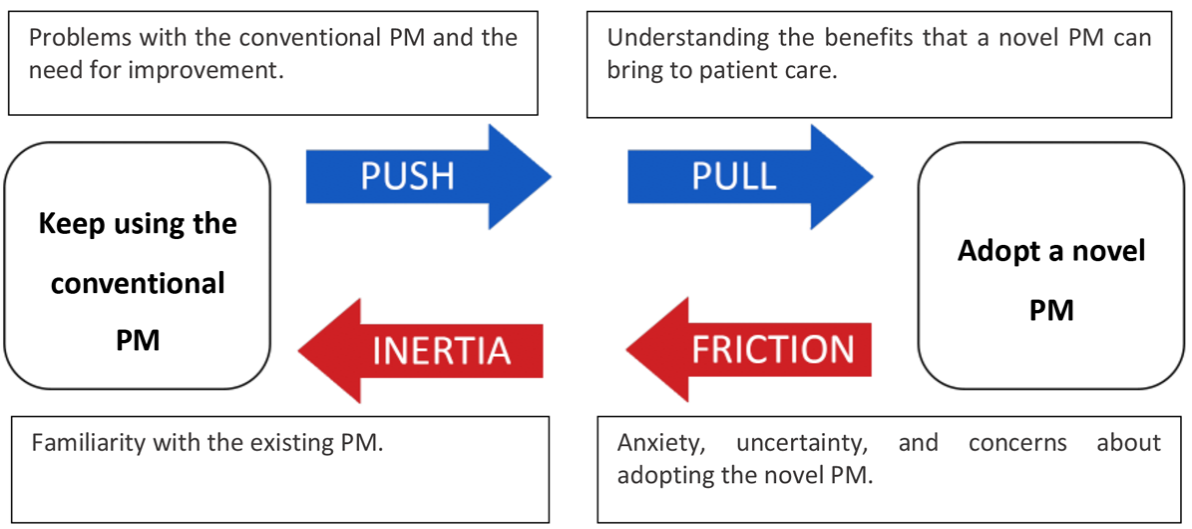
Balance of forces acting on the decision making of the clinicians when deciding whether to adopt a novel patient monitor for critical care or continue using the conventional patient monitor .

By fulfilling user requirements related to usability and SA, designers can significantly increase the chances of a novel PM being adopted by end users. However, there are natural barriers to the adoption of new technologies that need to be considered. For instance, familiarity with conventional monitoring tools and uncertainty about the novel PM are forces that contribute to the reluctance of clinicians to adopt a new approach. Therefore, for a new PM to be adopted, end users need to identify considerable benefits that the PM can deliver, in conjunction with a low burden of adoption [8]. Inherent in critical care medicine and PM design, in particular, is a high resistance to design changes by clinicians. This reluctance is based on their concern that changes to the status quo in terms of PM design can result in an increased risk of clinical errors [8]. This balance of forces, involved in the adoption of a new PM, is illustrated in Figure 3, which is adapted from a concept presented by Maurya (2017) in *The Science of How Customers Buy Anything* [9].

The specific aims of this paper are to review innovations in PM design proposed by researchers and to explore how clinicians responded to these new designs with a focus on usability and SA. The ultimate goal of this review was to review the design of new PM devices, designed to deliver improved usability and SA for nurses and doctors and hence the reduced likelihood of use error–induced risks to patients [10].

## Methods

### Article Selection

The literature search included data up to June 2019 with no cutoff on the start date. Search terms were chosen to reflect the review focus. The article selection was conducted in 2 phases: an initial search based on the Preferred Reporting Items for Systematic Reviews and Meta-Analyses (PRISMA) guidelines, followed by a search of the references within each of the previously identified papers. The PRISMA guidelines were used to identify relevant studies. The search was conducted with 7 relevant databases (Scopus, IEEE Xplore, PubMed, Science Direct, CINAHL, Cochrane Library, and Engineering Village) using the search terms presented in Textbox 1. Articles were further excluded after title, abstract, and full paper analysis by members of the multidisciplinary team. The papers included in this review were analyzed using a narrative synthesis approach.

**Search terms used in the database search. The search terms are grouped into 3 categories: patient monitor, usability, and hospital settings.**

Patient_Monitor: “patient monitor” OR “patient display” OR “vital sign* monitor” OR “vital sign* display” OR “monitor* display” OR “physiologic* monitor*” OR “physiologic* display”

AND

Usability: “human factor*” OR “usability” OR “ergonomic*” OR “human error” OR “UX” OR “user experience” OR “interaction design” OR “interface design”

AND

Hospital_Setting: “hospital” OR “intensive care” OR “ICU” OR “critical care” OR “operating room” OR “emergency department” OR “cardiology” OR “neurology” OR “oncology” OR “obstetrics”

### Inclusion and Exclusion Criteria

This review focused on the design and usability of prototype devices from research laboratories that were designed to overcome identified problems with commercial PMs. In this regard, the inclusion and exclusion criteria for this review were as follows:

- Studies published in English appearing in peer-reviewed academic sources.
- Studies that include user testing, comparing the performance and user experience of participants when using the novel prototype display and the traditional monitoring equipment. Studies that merely described the design of the prototype were not included in the review.
- The subjects participating in the experiment must be the intended users of the device (eg, ICU nurses or anesthesiologists). Studies in which participants were not the intended users (eg, undergraduate students) were not included in this review.
- The prototype display and the devices used as controls must be designed for real-time physiological monitoring. Therefore, novel prototypes that were designed specifically for trend and medical record analysis were not included.
- The prototype display must be a visual display designed for critical care use. Novel wearable prototypes such as tactile, head-mounted, and smartwatch displays were not included because this category of PM warrants a separate literature review focusing on wearable PMs. In addition, studies in which the focus was to test an enhanced algorithm with no meaningful enhancement on the user interface were not included.

The summary of the studies reviewed is presented in Multimedia Appendix 1. The selected studies were assessed regarding bias risk using an adaptation of the well-established Cochrane Collaboration tool for randomized controlled trials and crossover trials [11]. The results from the quality assessment are presented in Multimedia Appendix 2.

## Results

Table 1 provides a breakdown of the article search. The initial database search (including title, abstract, and keywords) yielded 136 articles. After the removal of duplicates and filtering by title, abstract, and full-text review, 10 items were included from the PRISMA search, and 5 additional items were identified during the reference search. Therefore, the final number of publications incorporated for review was 16. A summary of these publications is presented in Multimedia Appendix 1.

**Table 1:** Demographic characteristics of SEM survey respondents.

Database	Patient_Monitor search results	(Patient_Monitor search results) AND (Usability search results)	(Patient_Monitor search results) AND (Usability search results) AND (Hospital_Setting search results)
Scopus	11,720	249	69
PubMed	32,029	190	62
IEEE Xplore	131	4	1
Science Direct	3396	123	8
Cumulative Index to Nursing & Allied Health Literature	333	8	3
Cochrane Library	2928	14	8
Engineering Village	308	12	5
Number of publications identified	50,714	596	156
Remaining publications after removing duplicates	N/A^a^	N/A	136
Remaining publications after title assessment	N/A	N/A	83
Remaining publications after abstracts assessment	N/A	N/A	61
Remaining publications after full-text assessment	N/A	N/A	10
Additional publications found by references assessment	N/A	N/A	6
Publications included	N/A	N/A	16

^a^Not applicable.

### Graphical and Integrated Displays

Graphical displays (GDs) are designed to integrate the discrete vital signs from the PM into one or more multidimensional objects to facilitate improved assimilation by the clinician of the patient’s current state [12]. The concept seeks to take advantage of the natural human perception capability to detect changes in shape and color and use this capability as a means to convey relevant information effectively and efficiently. GDs and ecological displays (EDs) have been studied for complex, high risk, and data-rich environments such as commercial aviation control and power plant management [13,14] before the investigation of their use in health care.

Gurushanthaiah et al [15] performed one of the first studies to analyze the effect of GDs on patient monitoring performance. They did not develop a novel interface to enhance patient monitoring; rather, the authors tested 3 different displays that were available on a commercial anesthesia machine, the Ohmeda Modulus CD. The purpose of the study was to investigate with which display format anesthesiologists would perform better in terms of response time and accuracy. The displays tested were the numeric, histogram, and polygon displays. In each case, the displays monitored variables such as heart rate (HR), arterial blood pressure (Art), NIBP, blood oxygen saturation (SpO_2_), expired (end-tidal) partial pressure of carbon dioxide (CO_2_), and the percentage of inspired oxygen (O_2_).

**Figure 4:**
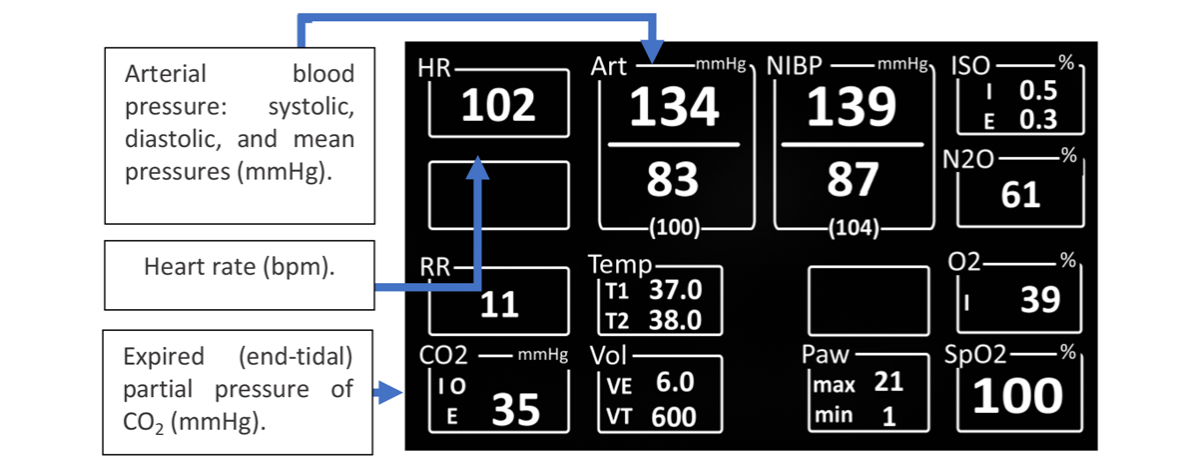
Numeric display (a model of the concept presented in the paper).

**Figure 5:**
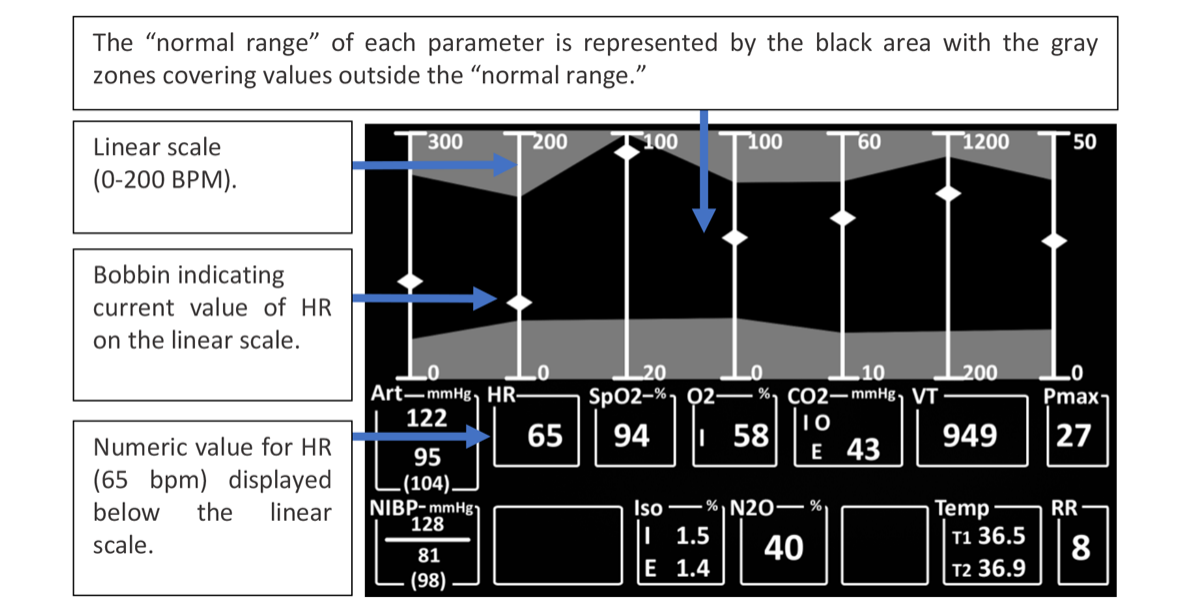
Histogram display (a model of the concept presented in the paper).

The numeric display (Figure 4) is considered a conventional display because each variable is presented in a numeric form using the single-variable-single-indicator approach, as used in a traditional PM. The main differences between this numeric display and the traditional PM are the arrangement of the variables, the presence of waveforms, and the lack of color-coding. Therefore, the user had to rely solely on the numbers and labels to assimilate the information. The histogram display also displayed the numeric values of the variables as in the numeric display; however, it also graphically presented the variables in the form of a *bobbin* sliding up and down on a linear scale as the value of the variable changed (Figure 5).

**Figure 6:**
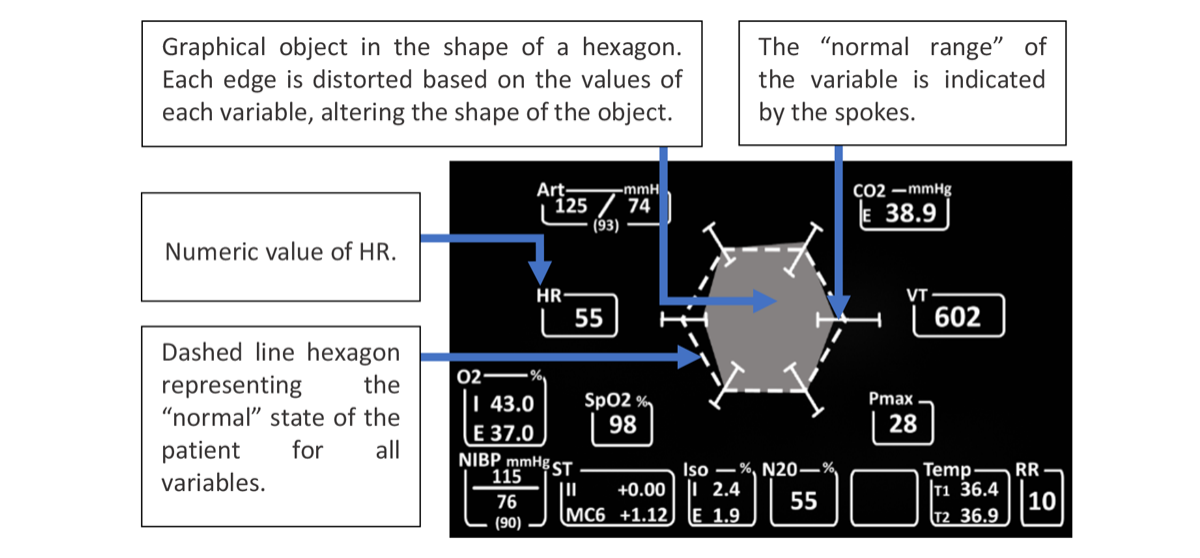
Polygon display (a model of the concept presented in the paper).

The histogram display depicted 7 variables in the form of scaled linear *tapes*, where a *bobbin* indicated the value of each variable on the vertical scale. The *bobbin* moved up and down proportionally on the linear scale as the value of the variable changed. The numeric value for each variable was also displayed directly below the linear scale (Figure 5). In addition, the normal range for each variable was represented by the dark region inside the graph. The polygonal display integrated 6 of the 7 variables (excluding O_2_), with each of the 6 variables forming a vertex of a hexagonal-type figure, occupying less space than the histogram graph. At each vertex of the hexagon, a bar indicated the maximum and minimum values reached by the parameter. As the variable changed value, the vertex moved along this bar. The dotted line indicated the *ideal* value for the variables; if the variable exceeded or was less than this value, then the vertex moved to a position where the resulting shape was a distorted hexagon (Figure 6).

Thus, the shape of the gray element in the display was indicative of the patient’s current state, and the users of the interface would be able to perceive the patient’s state based on the amount of deviation of the gray hexagon shape from the dashed line hexagon.

A total of 13 anesthesia residents were trained to use the displays and were asked to test the 3 different simulated data visualization formats. Participants were asked to indicate when they noticed a change in the variables and if the change was an increase or decrease in the variable value. It was observed that the response time and accuracy were significantly higher when the anesthesia residents used the graphic displays (histogram and polygon) in comparison with the numeric format. Although the order in which the displays were exposed to each participant was randomized, the randomization method was not detailed. This makes it difficult to judge whether the results were biased by carryover effects.

These positive results supported the use of GDs by anesthesiologists. However, within a few years, the polygon display option was removed from the next-generation Ohmeda Modulus CD anesthesia machine, as only a very small number of their customers used it. This finding motivated researchers to query the reason for the reluctance of clinicians to adopt this new approach. According to Drews and Westenskow [12], the difficulty of new displays in having to overcome user inertia could have contributed to the failure of the polygon display. This kind of inertia is a natural barrier to the adoption of new technology in critical care, where lives are at stake and users are more comfortable working with tried and tested interfaces. Another contributing factor may have been related to data visualization difficulties. To create a regularly shaped polygon when the patient’s state was normal, the spokes for each monitored variable had to be scaled at equal lengths. With this scaling, a significant change in one variable could be less perceptible than a significant change in another variable, thereby creating a risk of an anesthesiologist missing a critical event and putting the patient in danger [12]. This obvious usability problem highlights the importance of user testing with experienced end users who have a greater chance of flagging such problems before a device is released in the market.

**Figure 7:**
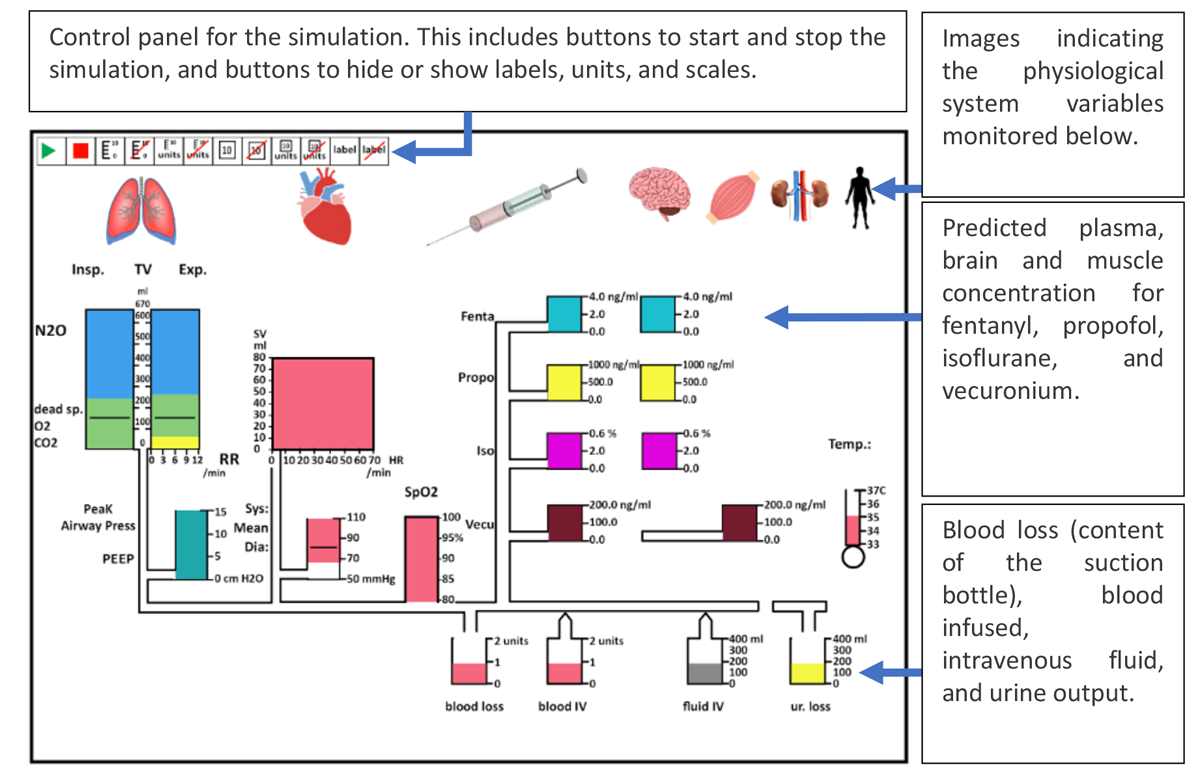
Michels et al (1997) display used to monitor 30 variables from a range of monitoring devices (a model of the concept presented in the paper). This display represented a patient in a normal state with all variables in acceptable levels including all labels, scales and units.

Michels et al [16] evaluated a custom-designed integrated GD (IGD), designed for anesthesia monitoring. The IGD (depicted in Figure 7) integrated not only the related variables from the same device but also data from different devices such as a PM, mechanical ventilator, and infusion pumps in a graphical manner.

**Figure 8:**
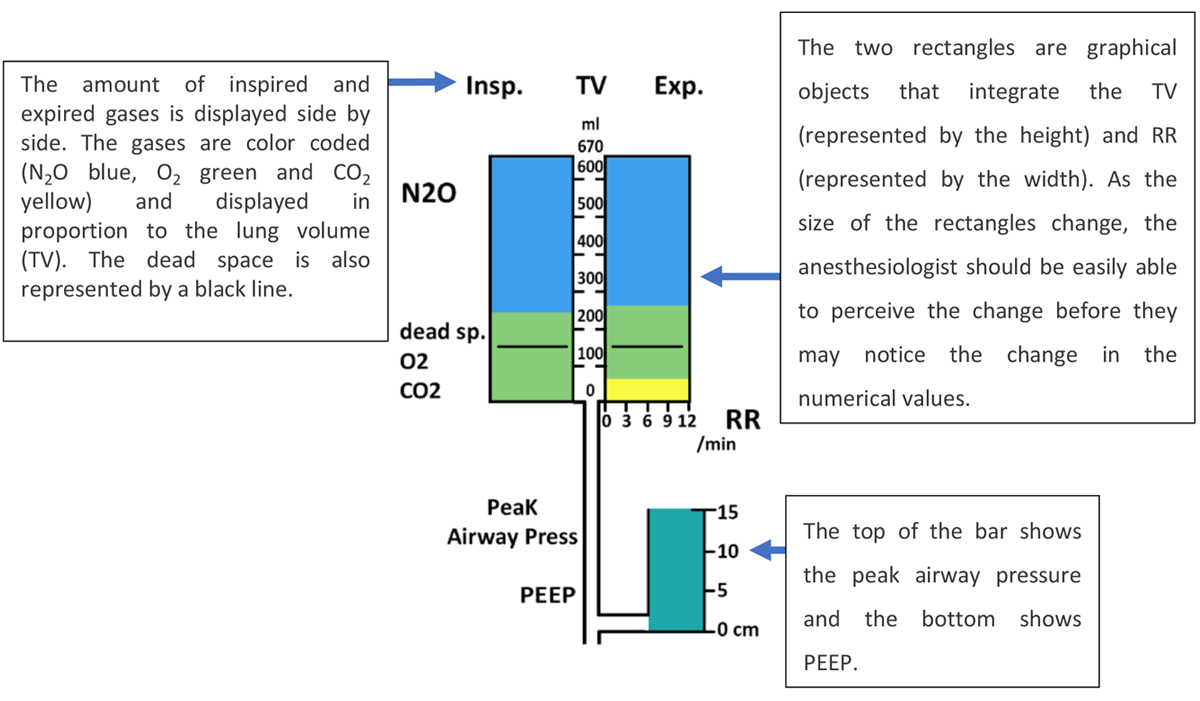
Respiratory system variables. The thresholds (represented by the black lines) for the vital signs and drug delivery indicating the acceptable levels for these variables.

**Figure 9:**
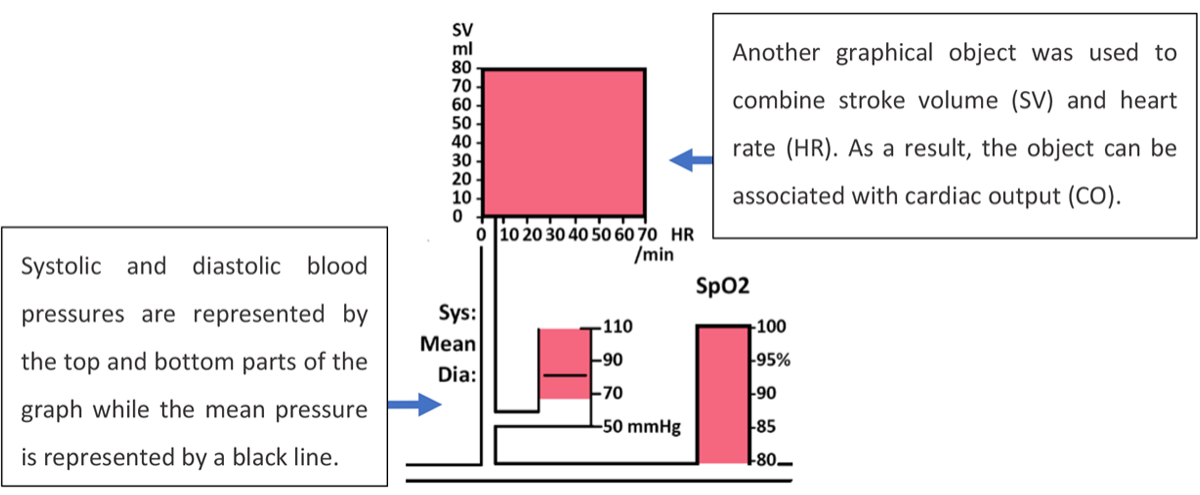
The cardiovascular system variables had the same colors.

On first exposure, this display may look overwhelming to the user because of the high number of variables presented on the display. To allow the user to interpret the display more efficiently, Michel et al [16] arranged the display elements from left to right based on the flow of gases and drugs through the body. The idea behind this strategy was to provide the clinician with an intuitive visualization that mapped the display element to the relevant human body system. The variables related to the respiratory system, such as inspired and expired tidal volumes, peak airway pressure, positive end–expiratory pressure (PEEP), and respiratory rate, were displayed on the left side, followed by cardiovascular, drug delivery, and fluid management variables toward the right of the display (Figures 8 and 9, respectively). In addition, color-coding was used for related variables, as shown in Figure 9.

**Figure 10:**
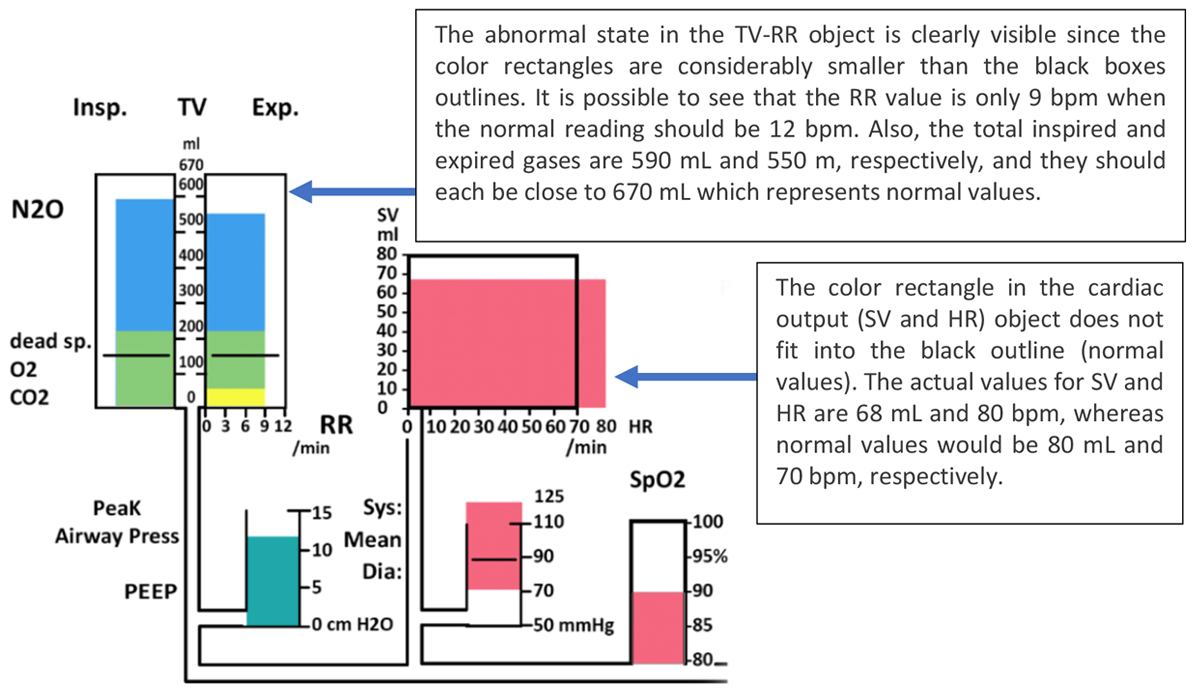
The display by Michels et al showing abnormal monitoring values in the respiratory and cardiovascular systems (a model of his concept).

The displays depicted in Figures 7 and 8 illustrate a patient in a healthy state. However, the levels of some variables could decrease or increase and exceed the threshold (vertically or horizontally). The anesthesiologist was able to detect the changes and abnormality of the parameters based on the distance of the actual levels of the variables from the threshold lines. The representation of the display by Michel et al [16] monitoring abnormal values is shown in Figure 10.

Ten anesthesiologists were asked to monitor a simulated patient in 4 different scenarios (blood loss, inadequate paralysis with spontaneous ventilation, cuff leak, and depletion of soda lime). Five anesthesiologists were asked to use the display by Michel et al [16], and 5 anesthesiologists used an anesthesia simulator (Body Simulation, Advanced Simulation Corporation) simulating a traditional PM. The results of the testing varied depending on the scenario used. For example, when participants used the IGD, the detection time was significantly shorter only for 2 scenarios (inadequate paralysis and cuff leak) and accurate event identification occurred significantly sooner only in 3 scenarios (blood loss, inadequate paralysis, and cuff leak).

This study demonstrated that IGDs have the potential to enhance the response time of anesthesiologists. The IGD presented in this study displayed all the information required by the anesthesiologists on a single screen, giving it an obvious advantage over conventional PMs under real-world conditions, where anesthesiologists would need to acquire information from multiple sources. For example, the anesthesiologist may have to ask the nurse to read the quantity of the blood collection bottle and measure the urine output.

The experimental design may have favored the IGD in this study as participants using the simulator in the experiment had to toggle through 4 screens on a single monitor to obtain the full range of clinical information, thereby influencing their response time with the simulator. This does not reflect the real-world conditions that the anesthesiologists would encounter, where all information would be simultaneously available on separate displays.

Another factor that might have affected the experiment was that participants from both groups were given a short introduction training session on the relevant display before the experiment commenced for approximately 15 min. Although all the questions were answered after the introduction, a short training session may not be sufficient to acclimatize clinicians to a completely new display, especially considering that the participants had never seen the IGD or used the body simulation system before.

**Figure 11:**
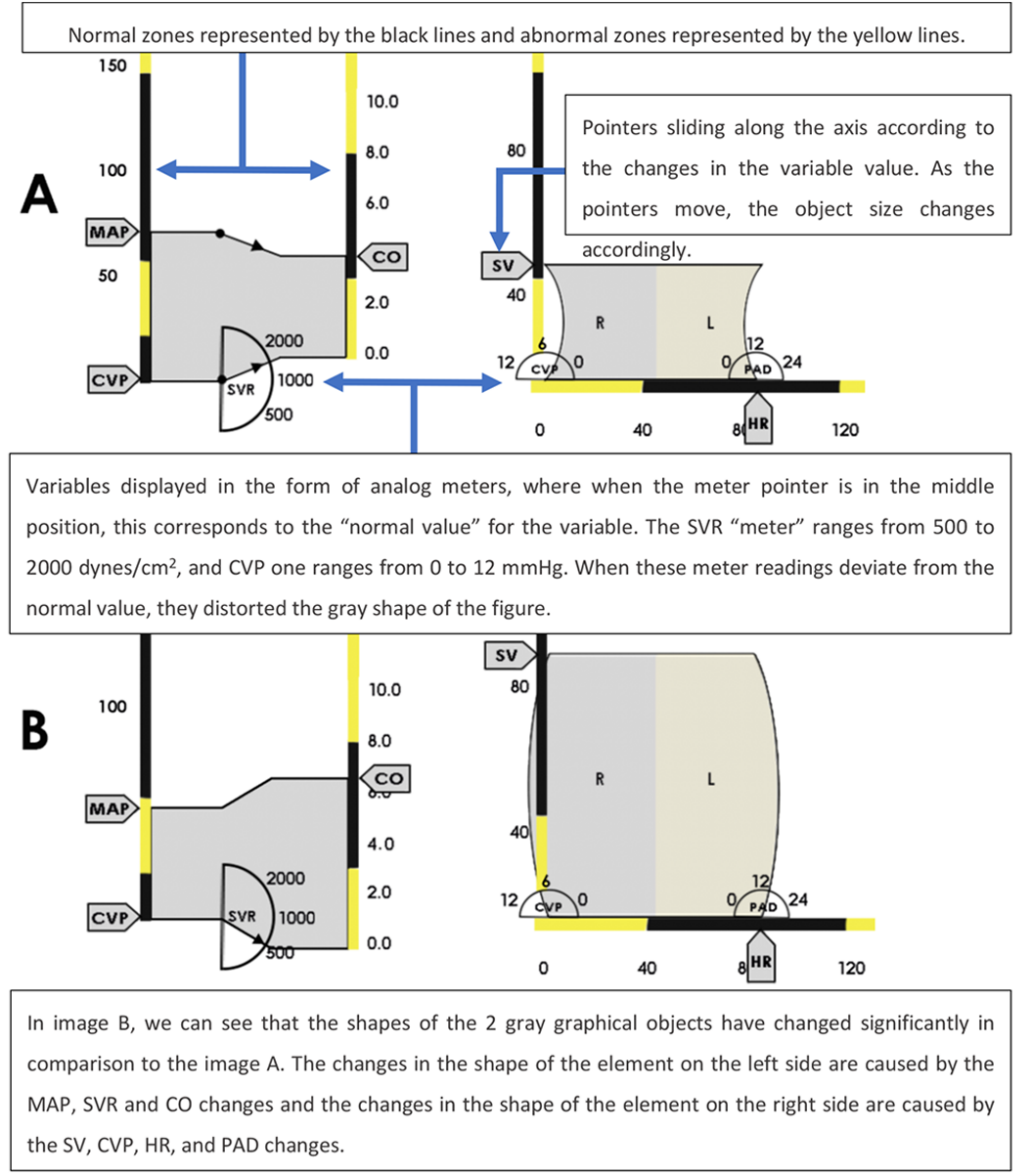
The graphical display by Blike et al contained 2 graphic objects that change shape and size depending on the changes in the values of the variables (a model of the concept presented in the paper).

Blike et al [17] developed and evaluated a cardiovascular GD designed to support anesthesiologists to perform a diagnostic task rapidly and correctly. Before the development of the display, the authors interviewed cardiac anesthesiologists to generate a decision model of how experts diagnose cardiac shock and determine its cause. Designers then developed the GD presented in Figure 11 based on the decision model created.

Blike et al [17] sought to improve the usability of their novel interface by arranging the elements on the screen in a meaningful manner. The GD was composed of 2 graphical objects, as shown in Figure 11. A new concept introduced by Blike et al [17] was the use of meters (gauge icons). In this concept, variables such as systemic vascular resistance (SVR), CVP, and diastolic pulmonary artery pressure (PAD) were presented in the form of meters with arrows indicating the values of these variables, with an arrow position at 12 o'clock, representing a normal value. Blike et al [17] compared the performance of this GD to an alpha-numeric display showing only the numeric values for blood pressure (BP), HR, CVP, PAD, and cardiac output (CO).

Using a between-subjects design, 11 anesthesiologists were presented with 10 scenarios (5 without cardiac shock and 5 with cardiac shock). Participants committed fewer diagnostic errors when using the GD in comparison with the alpha-numeric control display. The recognition of the patient's condition was also completed faster when using the GD. However, the authors reported that all participants used the control display first followed by the GD. This indicated a high risk of carryover effects, which could have contributed to biased results.

Interestingly, the authors reported that after a brief initial exposure to the GD, most participants expressed confusion regarding the display and “found it to be too complicated” [17]. Considering that Blike et al [17] brought new concepts to the display, such as the meters and graphical objects, it is therefore natural that such an innovative display would cause some level of discomfort for users on first exposure. As the use of the GD resulted in improved performance metrics according to the study, it would be interesting to know if extended exposure to this interface would be sufficient to overcome the reported negative initial impressions.

In a follow-up study, Zhang et al [18] compared the GD developed by Blike et al [17] with a commercial PM display. The study sought to investigate whether the use of the GD by Blike et al [17] could enhance the accuracy and response time of clinicians and whether it could also increase clinicians’ SA during the type of dynamic situation occurring in real practice. Zhang et al [18] developed 4 scenarios for the experiment: hypovolemia, arrhythmia, ischemia, and bronchospasm. Overall, 12 anesthesiologists (residents and faculty members) were asked to use the display by Blike et al [17] as the experimental display and a commercial PM (Datex AS/3 anesthesia monitor) as the control display. Participants were introduced to the new GD during the training phase. SA level 1 (related to the perception of the patient’s current state) and SA level 2 (comprehension of patient’s current state) were measured by routinely pausing the simulation and administering a questionnaire to the participant about the status of the variables displayed on the monitor. A higher number of correct answers indicated a higher level of SA.

The results showed that the anesthesiologists improved their detection time for the bronchospasm scenario, but no significant differences were found for scenario recognition time between the control and experimental displays. Level 1 SA was higher in the control condition during the arrhythmia, hypovolemia, and bronchospasm. Level 2 SA was higher for GD during the hypovolemia scenario. It is not clear whether the order of displays tested was randomized; therefore, it is not possible to confirm whether the results were affected by the carryover effect. In the same article, Zhang et al [18] presented the results from a second experiment involving a 3D IGD. However, insufficient information was provided in the study to fully understand the operation of this 3D GD, and the participants who tested the interface were not anesthesiologists; therefore, it was not discussed in this review.

**Figure 12:**
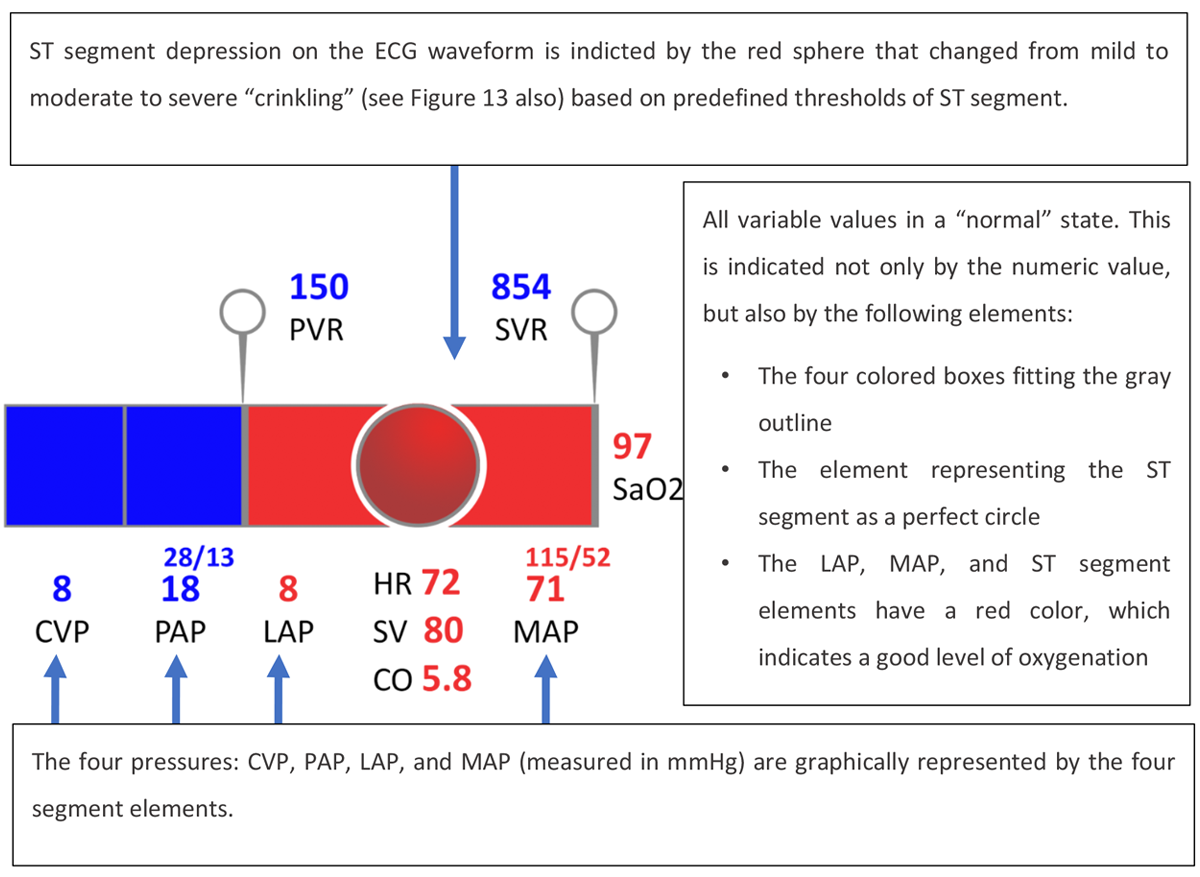
The cardiovascular graphical display by Agutter et al (2003) showing the vital signs of a patient in a normal state (a model of the concept presented in the paper).

**Figure 13:**
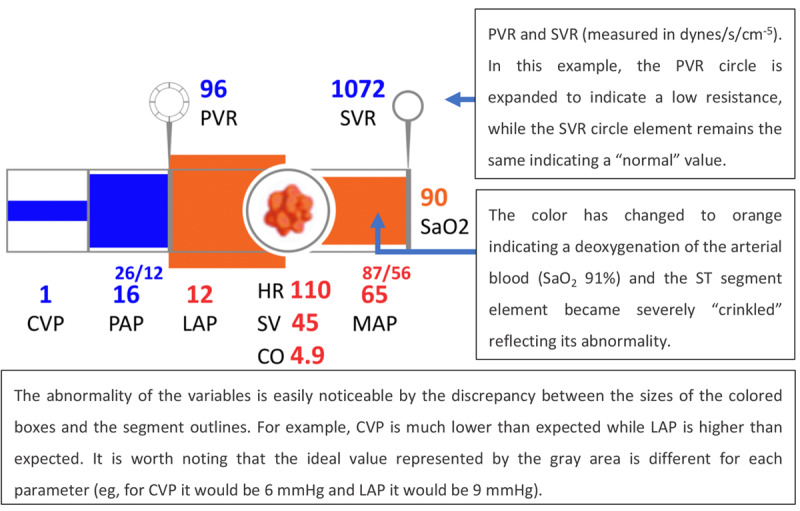
The cardiovascular integrated graphical display by Agutter et al (2003) showing the vital signs of a patient during myocardial ischemia (a model of the concept presented in the paper).

Agutter et al [19] developed a display designed for cardiology monitoring. The GD had the format of a 3D pipe, used as a metaphor for a blood vessel, as presented in Figures 12 and 13. Similar to the IGD by Michel et al [16], this GD also arranged the variables in a metaphorical manner to diagrammatically mimic physiological blood flow through the circulatory system. For example, central venous pressure (CVP) is the first element displayed as the deoxygenated blood flows to the vena cava. This blood flows through the pulmonary arteries to the lungs. Hence, the pulmonary artery pressure is displayed next in the sequence. After oxygenation, the blood flows to the left side of the heart and is then pumped into the aorta. Therefore, left atrial pressure (LAP) and mean arterial blood pressure (MAP) are the elements in the sequence. Other variables monitored by the display include pulmonary vascular resistance (PVR), HR, stroke volume (SV), CO, SVR, and arterial blood oxygen saturation (SaO_2_).

Numeric values for each variable were presented directly below their respective segment, and the height of each segment was directly related to its value. The oxygenation level (SaO_2_) was indicated by the color change from deoxygenated (blue) to oxygenated (red) after passing through the lungs.

**Figure 14:**
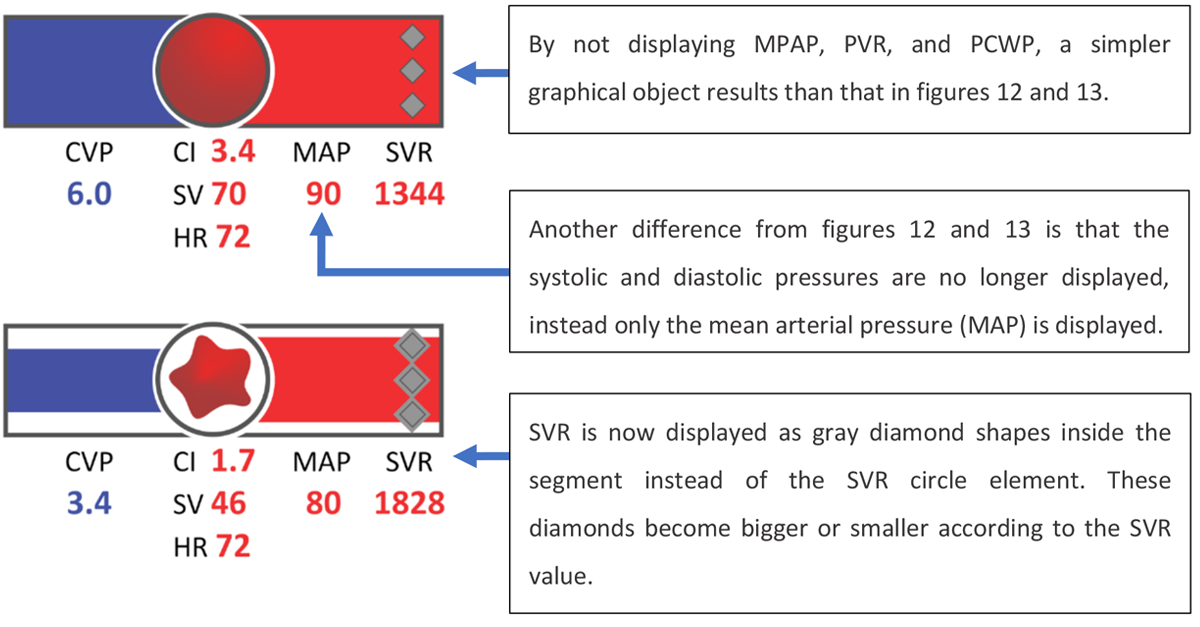
The integrated graphical display by Albert et al (2007) without pulmonary artery catheter data (a model of the concept presented in the paper) displaying the patient in a normal state and during myocardial ischemia.

A total of 20 anesthesiologists were invited to participate in the testing and were asked to assume care of a simulated patient (an instrumented mannequin connected to the monitor) in a high-fidelity simulation. Of them, 10 participants used GD as the experimental display and 10 participants used a numeric monitor, showing real-time values for the same variables appearing on the GD, as the control display. In addition, both groups used a commercial PM (Datex AS/3 monitor) in its full operating mode. Two scenarios were developed for the experiment: (1) total hip replacement with a transfusion reaction to mismatched blood and (2) a radical prostatectomy with 1.5 liters of blood loss and myocardial ischemia. The results show that participants using the GD could detect and treat ischemia faster than participants using the control display in the second scenario. It was also observed, for each scenario, that participants who used the GD finished the scenario with CVP and SaO_2_ values closer to the baseline values than participants using the control display. In the first scenario, participants did not detect the anaphylaxis faster, as expected, with the authors observing that changes in SVR and PVR could have helped in making this diagnosis. However, the changes in these display elements were not noted by the participants. This led to a redesign of these elements to improve their salience (as presented in Figure 14). In this study, the authors commendably strived to create an environment and context of use as close to real-world conditions as possible, in contrast to some of the other studies reviewed in this study. This led to important problems with the display being uncovered, allowing designers to solve the interface deficiencies that led to use errors.

As this GD was designed to be used in conjunction with a commercial PM, as an additional screen in the OR, it was important to investigate whether this new information source could affect the clinician's workload and mental demand. Participants were asked to answer a NASA Task Load Index (NASA-TLX) questionnaire, which is used to evaluate the self-perceived workload. Although participants had only a brief introduction to the GD before the experiment (approximately 15 min), the authors did not report significant differences in the workload ratings between the GD and control displays. This indicates that the novel display was successful in conveying information without imposing additional physical or mental demands on the clinician.

As a follow-up, Albert et al [20], from the same research group, evaluated the display developed by Agutter et al [19]. The rationale for this experiment was that, despite the positive results in the experiment by Agutter et al [19], regarding the time to diagnose and treat myocardial ischemia, Albert et al [20] identified some limitations in the experiment by Agutter et al [19]: (1) the IGD was evaluated in only 2 scenarios, (2) investigators recording the participants’ actions were not blinded to the presence or absence of the IGD, and (3) the display by Agutter et al [19] required the use of a pulmonary artery catheter (PAC) to obtain the CVP, pulmonary capillary wedge pressure, cardiac index, and SVR values, when it is not a part of routine monitoring for most anesthesiologists. The purpose of this new study was to address these limitations and broaden the applicability of the display, presenting it in 2 formats: with and without PAC-derived data. The representation of IGD without PAC-derived data is shown in Figure 14.

A total of 16 anesthesiologists and anesthesia residents participated in the new evaluation, 8 participants in the intervention group (using a commercial PM and the GD) and 8 in the control group (using a commercial PM and only the numeric values from the GD). Six scenarios were developed for the experiment: 3 without PAC-derived data (hypertension because of inadequate analgesia, myocardial ischemia, and hemorrhagic hypovolemia) and 3 with PAC-derived data (left ventricular failure, septic shock, acute respiratory distress syndrome, and myocardial ischemia). Two experts were invited to rate the participants’ performance from best (rank 1) to worst (rank 16) in terms of accuracy, timeliness, and quality. Unlike in the experiment by Agutter et al in 2003 [19], in this case, the experts were blinded to the display used by the participant, which reduced the risk of detection bias.

**Figure 15:**
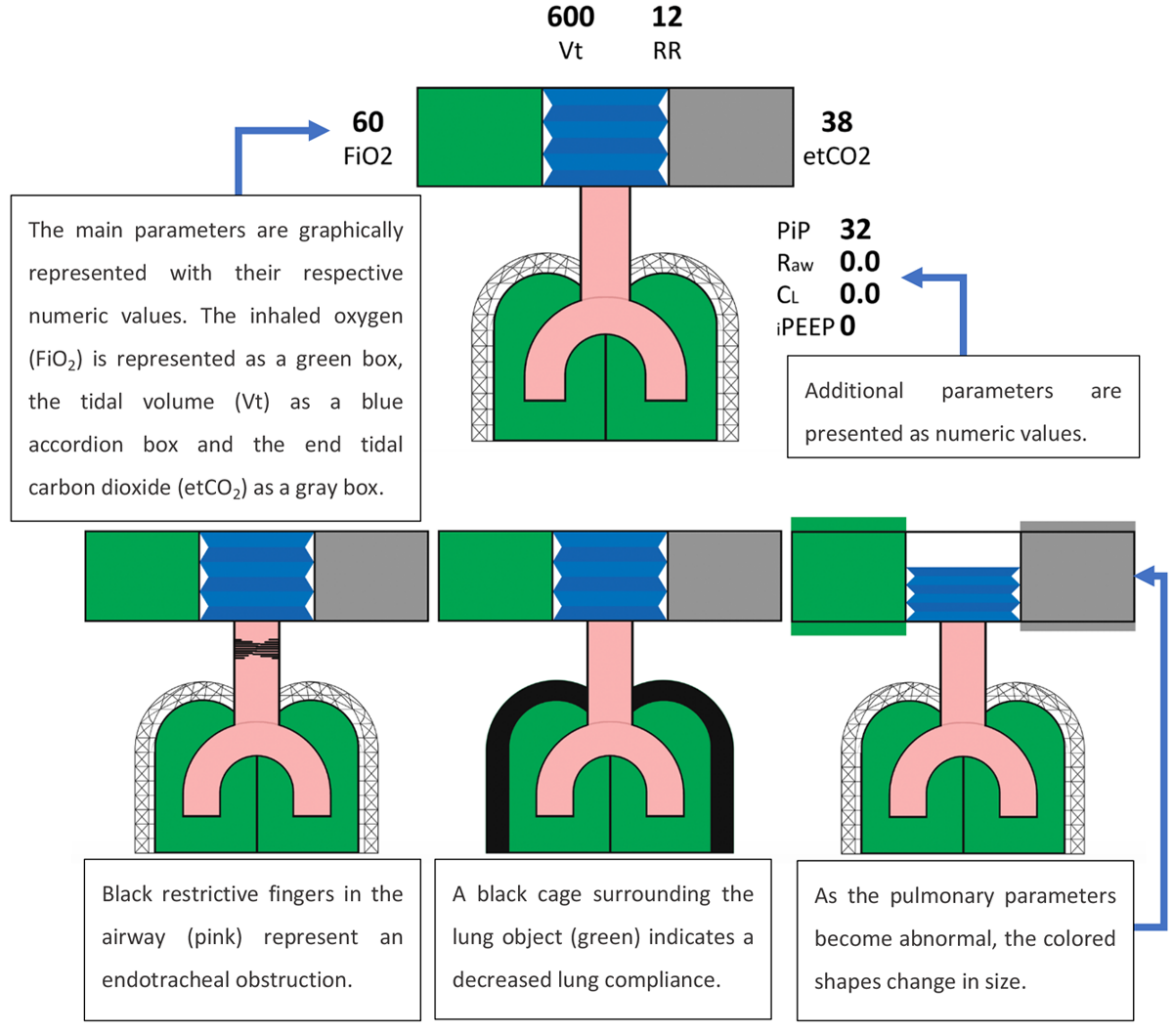
The figure at the top depicted pulmonary graphical display in which pulmonary variables are within the normal range. The design included a graphical display and numeric values. Examples of abnormal pulmonary variables are represented at the bottom (a model of the concept presented in the paper).

Wachter et al [21] developed a GD that presented the respiratory parameters for patients who were intubated and mechanically ventilated. The pulmonary GD displayed the parameters by making use of the anatomical shape of the lung (Figure 15). A total of 19 anesthesiologists, split into control and intervention groups, were asked to assume care of a simulated patient midway through a surgical procedure in a simulated OR. The simulation was composed of conventional monitoring equipment (a traditional PM), an anesthesia machine, and a cart containing airway management equipment. Both groups had access to the standard displays, but the intervention group also had access to the pulmonary GD on a 17-inch monitor.

Two expert anesthesiologists assessed participant performance. It was found that when using the pulmonary GD, participants detected and treated 2 out of 5 scenarios (obstructed endotracheal tube and intrinsic PEEP) significantly faster and reported lower subjective workload than when using the conventional monitoring setup. In addition, the accuracy of the participants was significantly higher in the intrinsic PEEP scenario when using the GD. However, in 2 scenarios (endobronchial intubation and hypoventilation), the number of incorrect diagnoses was higher (not significantly) with participants using the pulmonary GD.

Participants using GD in scenarios involving mild pain, myocardial infarction, and left ventricular failure were rated higher in performance than participants in the control group. In addition, participants using the GD detected and treated myocardial ischemia faster than those who did not use the GD. Once again, there was no statistically significant effect of the GD on the self-assessed workload as measured by the NASA-TLX.

**Figure 16:**
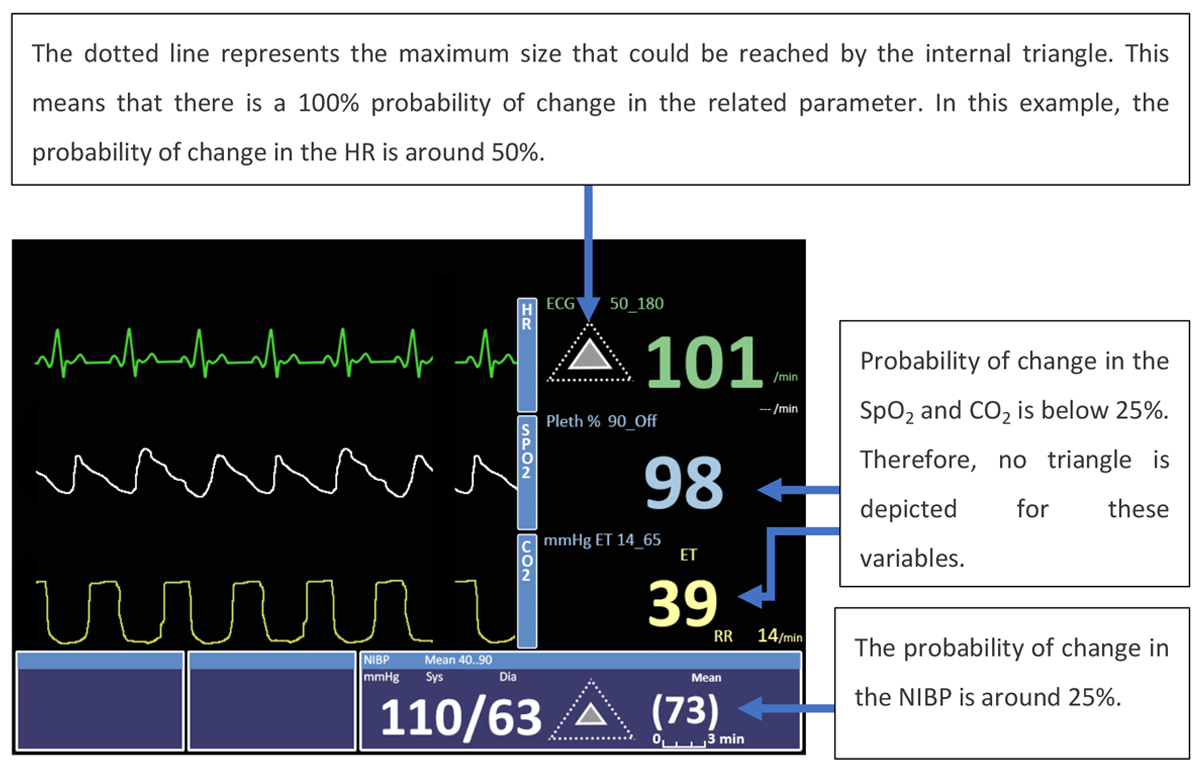
The enhanced display (a model of the concept presented in the paper) by Tappan et al (2009). The visual cue was a triangle object placed between the waveform and numerical values, which were displayed as in a traditional patient monitor. The size of the triangle changed according to the probability of change for each variable.

Tappan et al [22] explored the hypothesis that the simple addition of a graphical visual cue to an existing traditional PM (rather than a complete redesign) would be sufficient to improve the detection ability and response time of a clinician to a change in a patient variable. The display tested was almost identical to a traditional PM, with the only difference being the incorporation of a triangle between the waveforms and the numerical values (Figure 16). The size of the triangle would change according to the probability of change (increasing or decreasing) for each variable. When the probability of a change in the variable was below 25%, no triangle was displayed. If it was above 25%, the triangle was displayed to attract the attention of the observer. If the probability of change went beyond 25%, the triangle became proportionally larger. Along with the triangle, an outline of the maximum possible size of the triangle was also displayed as a reference. The display was compared with a simulated PM in terms of detection time and the number of events missed.

A total of 22 participants (anesthesiologists and anesthesia residents) were asked to identify when a change occurred in the monitored variables using the enhanced display and the control display, which consisted of the same display without the graphical visual cue. The detection time was reduced on average by 14.4 (SD 12) seconds when using the PM with the graphical visual cues when compared with the traditional PM. The percentage of missed events was 11.2% when using the PM with the graphical visual cues and 18.8% when using the traditional PM. A usability questionnaire was applied, but no significant differences were found regarding satisfaction between the 2 displays. These results show that to improve the performance of PM users, a complete redesign of a commercial PM is not always necessary. However, it is important to keep in mind that the usefulness of the display is dependent on the accuracy of the algorithm that calculates the variable change. If the algorithm is not accurate or is not perceived as accurate by the PM users, this change in the PM may generate frustration, leading to a negative impact on patient care.

The GDs described so far in this review were designed to support the needs of anesthesiologists in the OR, taking into account their decision-making process [17,18] or the biological mapping of vital signs [16,19,20]. However, another important user of PMs that must be taken into account when designing a new PM is the nurse, as *clinical monitoring by a vigilant nurse is the basis of intensive patient care* [23].

Görges et al [24,25] described 2 integrated displays where they combined numeric values, trends, alarm status of vital signs, infusion pump information, and therapy support indicators into 1 screen. The displays were designed to support ICU nurses and doctors when they have to quickly choose which patient to treat first from a distance of 3 to 5 m. For this reason, these displays were referred to as far-view displays.

**Figure 17:**
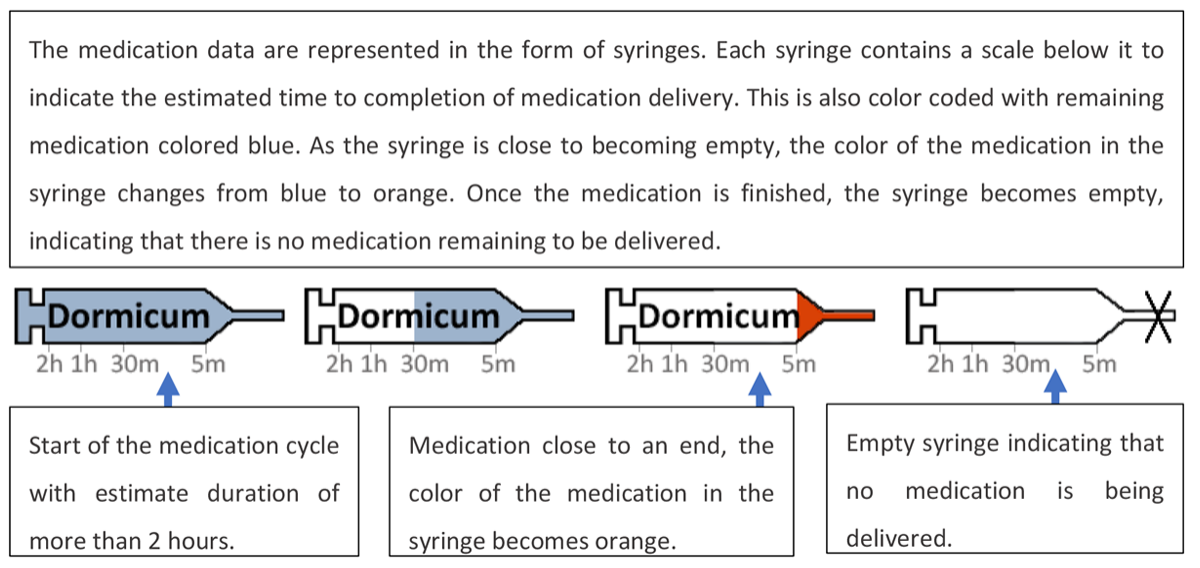
Four stages of drug delivery represented by the syringe by Görges et al (2011, 2012).

**Figure 18:**
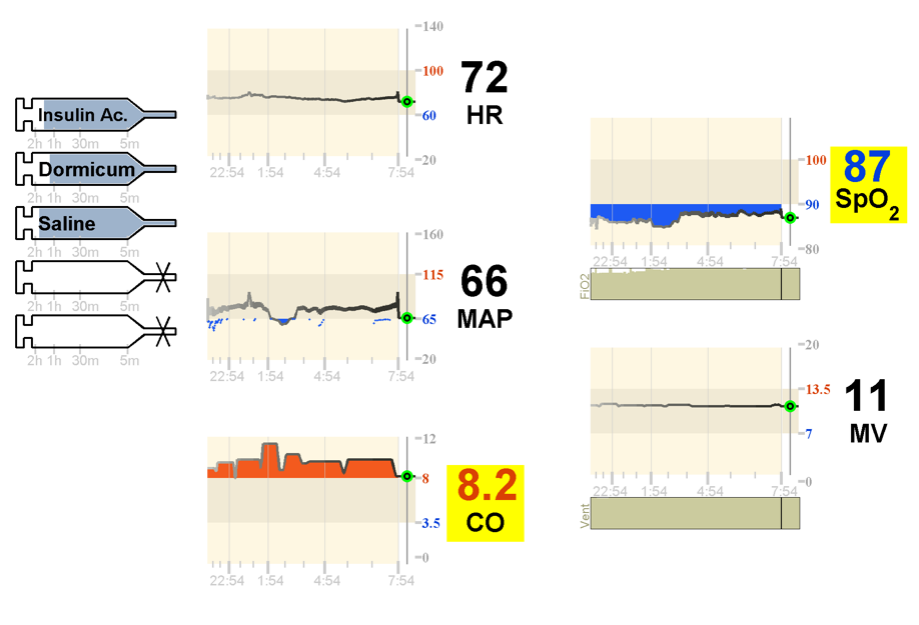
Integrated trend display tested by Görges et al (2011, 2012).

**Figure 19:**
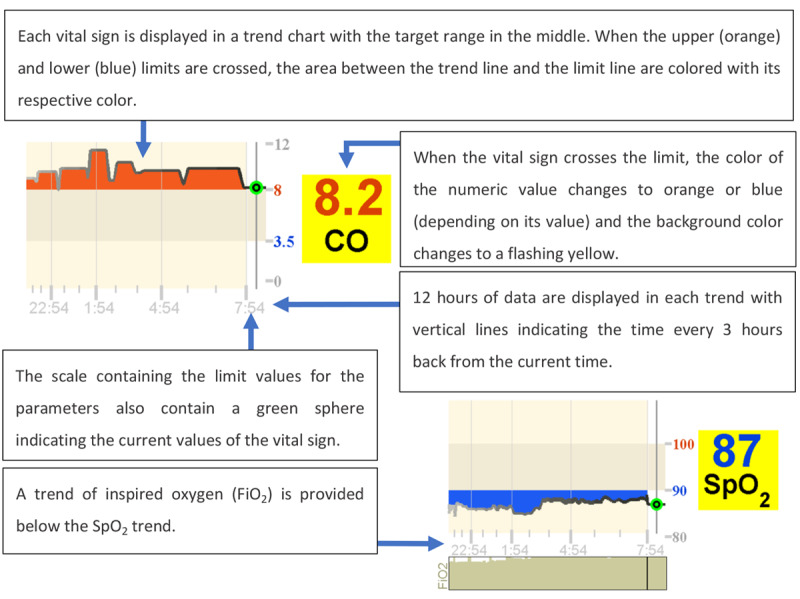
The trend element in Görges et al (2011, 2012) far-view bar display.

On the left side of the display, the displayed images of syringes indicated which medicine the patient was currently receiving and how long it would take for full delivery of the medication to be completed as illustrated in Figure 17. The display presented in Figure 18 is referred to as a far-view bar display. On the middle and right sides of the display, 5 variables were monitored using trends: HR, MAP, CO, SpO_2_, and ventilation minute volume (MV). Each graph was composed of a 12-hour trend highlighting the target zone for the variable and a numeric element depicting the current value of the monitored variable. The trend element in this display is shown in Figure 19 [24,25].

**Figure 20:**
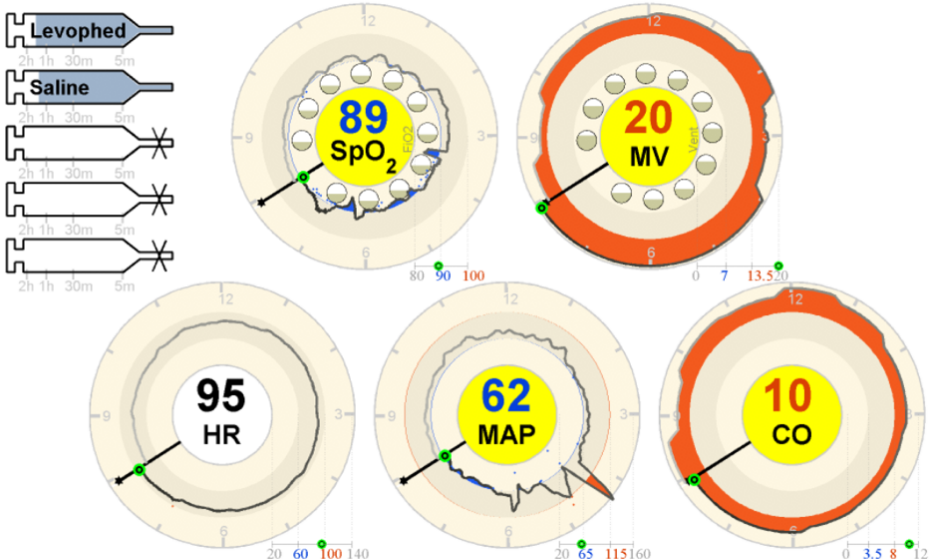
Integrated clock display tested by Görges et al (2011, 2012).

**Figure 21:**
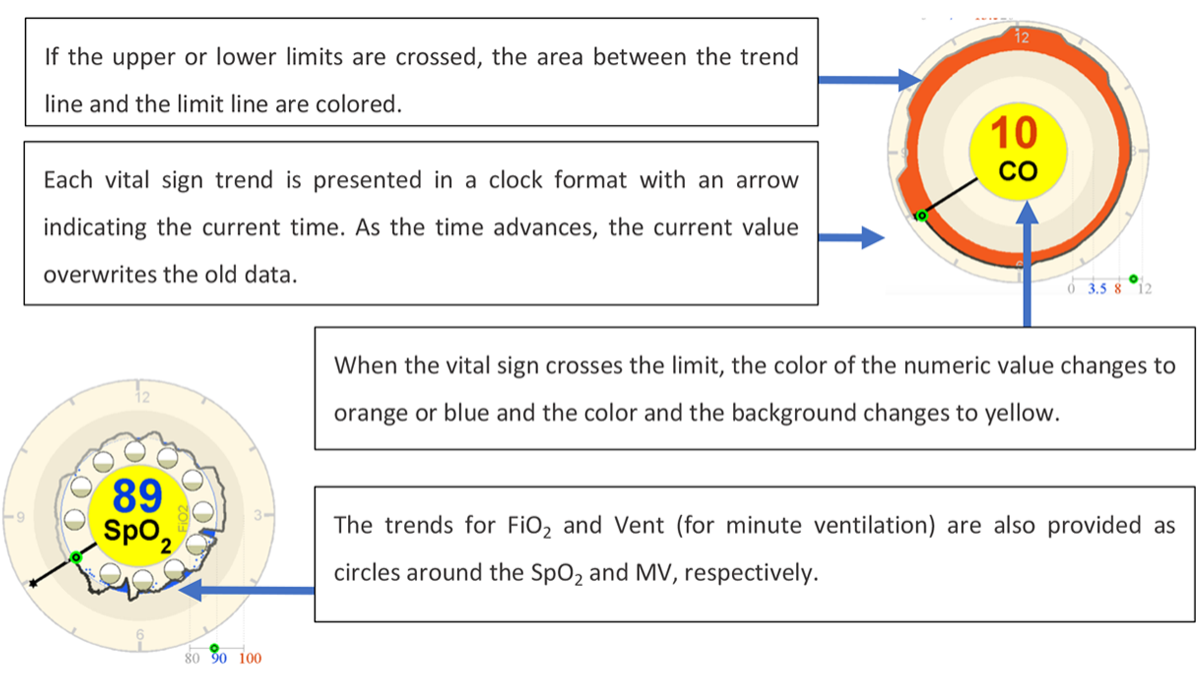
The clock element in Görges et al (2011, 2012) far-view clock display (a model of the concept presented in the paper).

The display presented in Figure 20 is referred to as a far-view clock display. It displays the same data as the bar display in a circle that looks like a clock in which the new variable values overwrite the old ones after 12 hours. The clock element in this display is explained in detail in Figure 21. The values for inspired oxygen (FIO_2_) and MV were presented within the circle using 12 circles (1 for each hour) instead of trends, with the current values being the background for the SpO_2_ and MV, respectively.

In both the studies (2011 and 2012) [24,25], participants were asked to take care of 2 patients simultaneously and decide which of the 2 patients required attention first, based on the information provided on the display. In the intervention condition, participants were using the integrated displays, and in the control condition, participants were using a commercial PM (Draeger Kappa XLT PM) and 4 commercial infusion pumps. In the first experiment, involving 16 ICU nurses, it was found that the decision time was shorter and the accuracy was higher when using the 2 novel displays. The results from the NASA-TLX questionnaire indicated that both far-view displays performed statistically significantly better than the control PM in terms of self-perceived frustration. Interestingly, more than half of the participants (n=9) preferred conventional displays. Unfortunately, these participants were not asked why they preferred the conventional displays. A particular feature that all nurses liked from the integrated display was the addition of the syringe functionality.

In the second experiment, 15 ICU physicians performed the same task. The physicians made more appropriate decisions and took less time in deciding which patient required attention first, when using the 2 novel displays. No statistically significant differences were found in the clinician workload when using the 3 displays. Regarding preferences, 1 physician preferred the control display, whereas 10 preferred the bar display and 4 preferred the clock display. Once again, participants were not asked the reason behind their preference, which makes it difficult to understand why nurses and doctors differed in their preferences.

Koch et al [26] conducted a thorough investigation of the tasks performed by ICU nurses, intending to provide recommendations for the design of integrated PMs, which could enhance the SA of nurses. In this study, 19 ICU nurses were observed for 38 hours in 3 clinical practice settings. The team wrote extensive field notes that were classified into 46 distinct tasks. These tasks were then grouped into categories for communication, medication management, patient awareness, organization, and direct patient care.

Koch et al [26] identified that essential information was deemed to be missing at the bedside, and even when the information was present, it was not integrated at the task level. Using the concepts presented by Endsley [7], Koch et al [26] classified the challenges arising from this lack of integration as perception, comprehension, and projection challenges. On the basis of the identified information gaps, Koch et al [26] provided recommendations for enhancing SA for frequently carried out tasks. These recommendations included (1) establishing methods of information sharing from any location, (2) an integrated display inside the patient’s room containing all the information necessary on 1 screen, and (3) making the relevant information visible and readable from the doorway.

**Figure 22:**
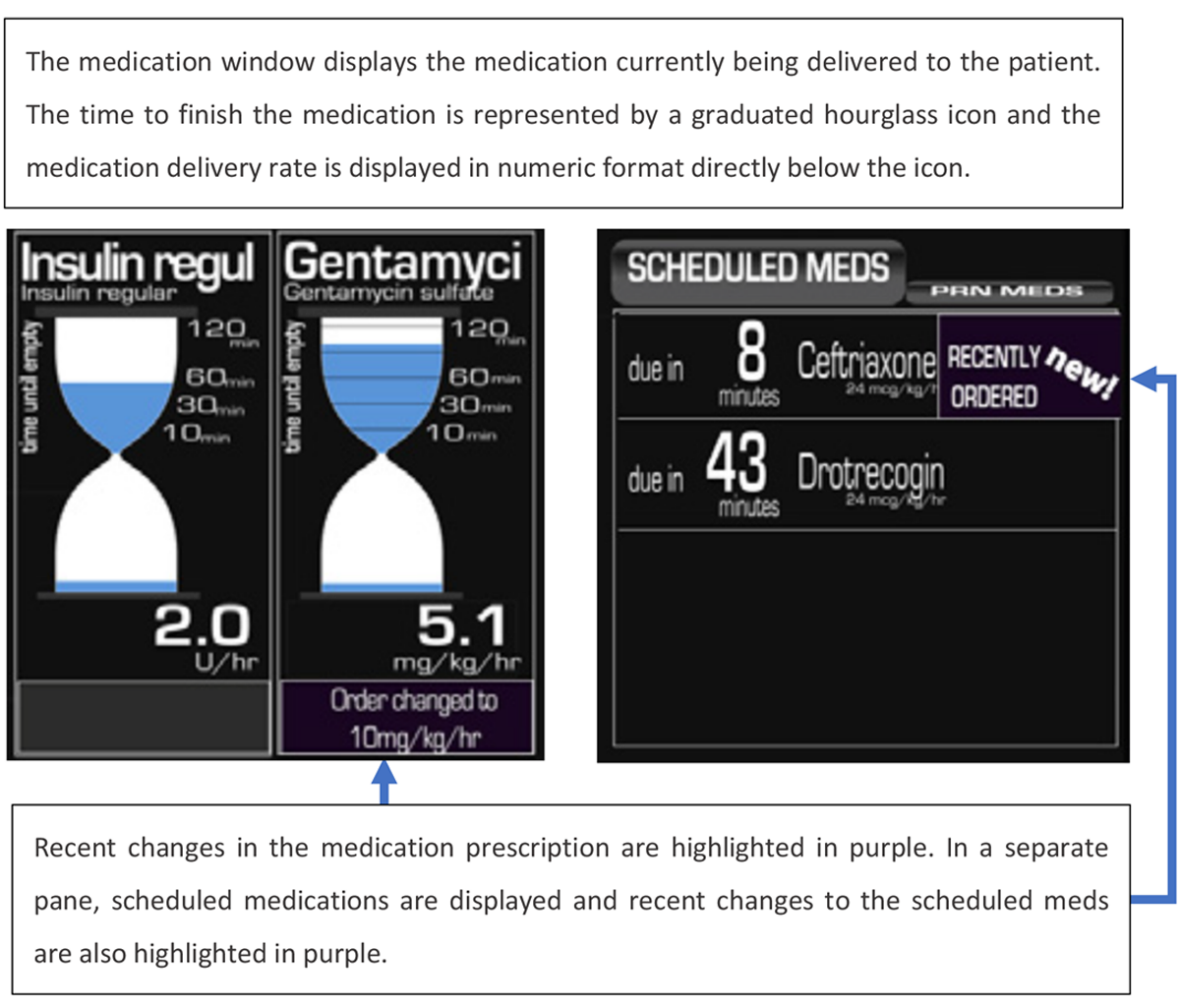
Koch et al (2013) medication windows added to the integrated display.

As a follow-up to this investigation, Koch et al [27] developed a paper prototype of a new integrated display. In contrast to the displays by Görges et al [24,25], the display by Koch et al [27] did not make significant changes to the look and feel of the display, when compared with a traditional PM. The waveforms and numerical values were displayed as in a traditional PM, but some elements from an even wider range of medical devices were added to the screen. For instance, ventilator settings, fluid balance, and temperature data were also included as numeric values below the vital signs, and the scheduled and current medications were displayed on the right side of the display. The medication windows are shown in Figure 22.

**Figure 23:**
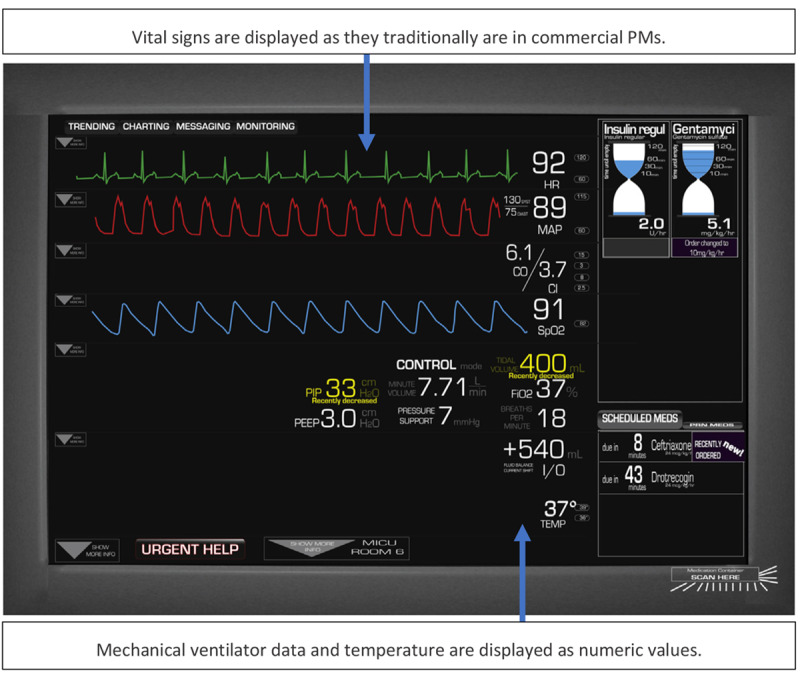
Koch et al (2013) prototype of an integrated display. The display shows scheduled and current medication, vital signs, ventilator settings, fluid balance and temperature.

In the study by Koch et al [26], it was established that most tasks performed by nurses relate to medication management, patient awareness, or team communication. Therefore, 3 common scenarios for nurses interacting with information systems were developed to cover each of these 3 aspects. A total of 12 nurses from a burn trauma ICU were asked to use 2 paper-based prototypes (the order of the displays was randomly assigned): (1) the new experimental integrated display (Figure 23) and (2) the screens from each device separately (not integrated). It was found that the SA (represented by the accuracy of the participants’ answers to questions asked during the testing) was higher, and the task completion time was shorter when using the integrated display.

This study demonstrates that the integration of data from multiple devices does not always require a radical change in the look and feel of the conventional PM. In a number of the studies reviewed thus far, we have seen that complete PM interface redesigns can lead to resistance from clinicians for reasons already discussed. Nonetheless, additional experiments using high-fidelity prototypes are required to ensure that the new design is useful and would be adopted by the users in critical care.

**Figure 24:**
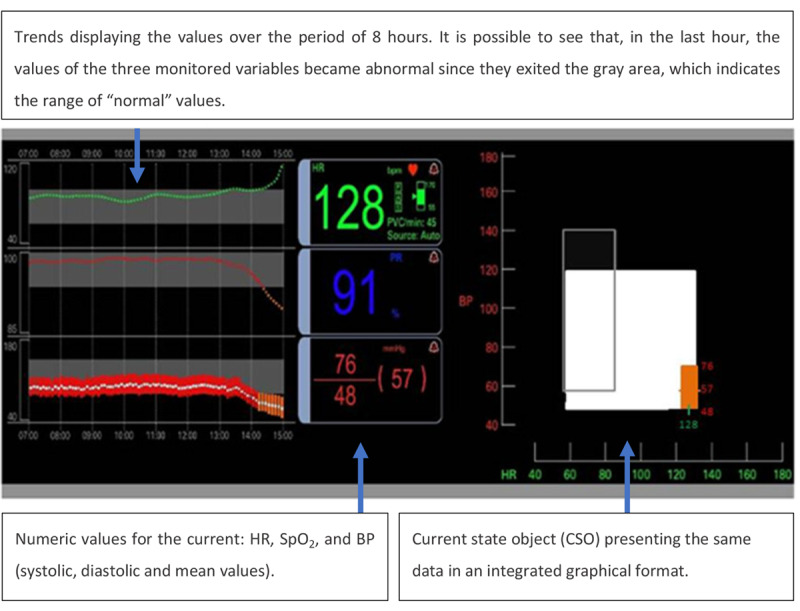
Drews and Doig’s graphical display. On the left side, data were presented in a similar manner to a traditional patient monitor, but with trends instead of waveforms of the vital signs.

Drews and Doig [28] developed a GD to support rapid detection and identification of physiological deterioration in patients by ICU nurses. This display was developed with a focus on ICU nurses’ needs and to address areas of improvement in commercial PMs identified in previous studies [29,30]. The interface was developed using an iterative design process with 3 experienced ICU nurses evaluating the display after each iteration. As shown in Figure 24, the GD monitored HR, SpO_2_, and BP. It was composed of 3 main components: trend data, numerical data, and a graphical object.

**Figure 25:**
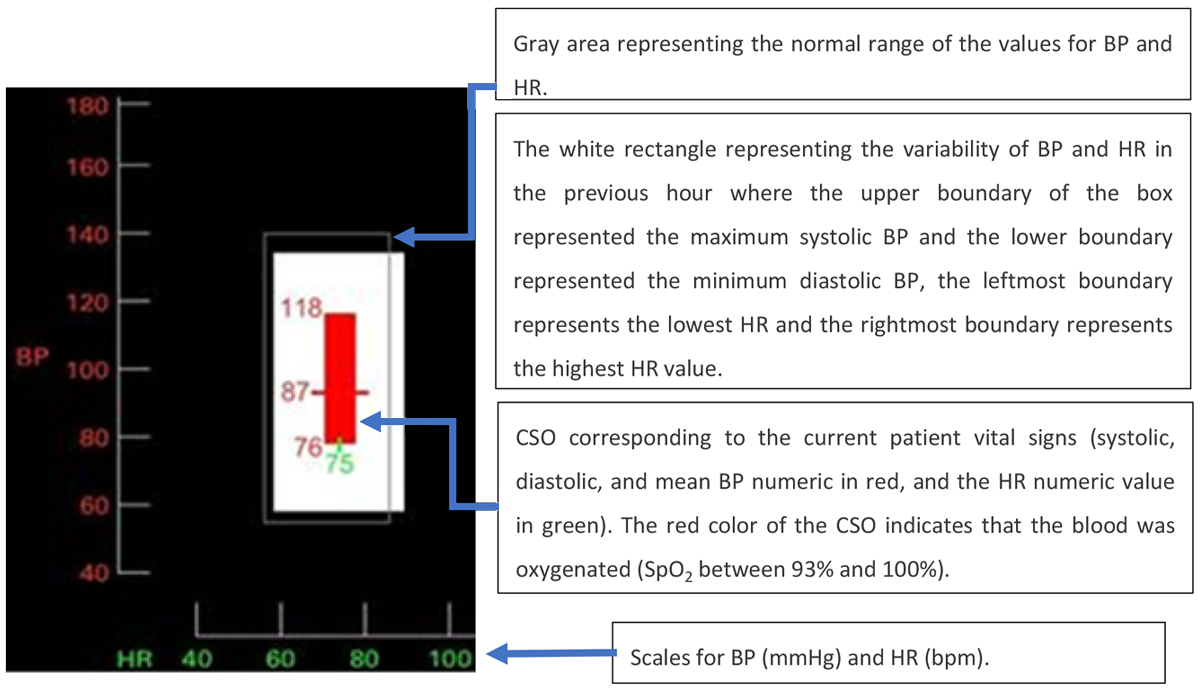
The graphic object combined the blood pressure and heart rate values to create an object that depicts the current state of a patient.

For each variable, the trends displayed the values from the previous 8 hours on a line graph. The line graph contained a gray area representing the normal range of the values. The numerical data corresponded to the current values of the variables. The current state object (CSO), explained in detail in Figure 25, combined HR (in the X-axis) and BP (in the Y-axis). The white rectangle represented the variability of BP and HR in the last hour, where the upper boundary of the box represented the maximum systolic BP, the lower boundary represented the minimum diastolic BP, the leftmost boundary represented the lowest HR, and the rightmost boundary represented the highest HR value. The gray rectangle represented the normal or customizable thresholds, and the colored element inside (or outside) the white rectangle represented the current patient vital sign measurements. The color reflected the SpO_2_ level, which could be red (93%-100%), orange (91%-92%), pink (89%-90%), purple (87%-88%), or blue (<87%).

The GD was compared with a simplified version of a PM (control) in terms of response time and accuracy of data interpretation. The simplified version of the PM contained only a numerical display, as presented in Figure 24, without trends or CSO. In both conditions (intervention and control), the vital signs were also displayed on a desktop computer along with the display being tested. Four scenarios were developed for this experiment: early sepsis, septic shock, pulmonary embolus, and a stable scenario. On the basis of the provided display and context information, 42 ICU nurses (21 using the novel display and 21 using the control display) were asked to evaluate and interpret the data and recommend appropriate interventions as quickly and as accurately as possible.

Overall, the participants using the GD were 30% faster than participants using the simplified traditional display, with statistically significant differences for septic shock, pulmonary embolus, and stable vital sign scenarios. In terms of accuracy, participants correctly identified the condition of the patient with statistically significant differences in septic shock and pulmonary embolism scenarios. A NASA-TLX questionnaire distributed after the test revealed a statistically significant difference in the mental demand, with lower mental demand reported by nurses using the GD.

The purpose of this experiment was to measure the performance of the nurses when using a single-sensor-single-indicator display compared with a graphical or object display. In this sense, it is understandable that the presence of waveforms on the control display was not essential. However, because the novel display was designed to replace the conventional PM display, it is unusual that the control display did not adopt the full PM interface in daily use by the end user. This theme of so-called control displays not truly representing the display used by users in their everyday work, recurs throughout some of the studies presented in this review.

### Ecological Displays for Patient Monitoring

Some authors have used a framework for interface development called ecological interface design (EID). EDs attempt to minimize the cognitive load on the user by presenting data in a meaningful way, depicting the relationship between data elements and making the constraints of the monitored system visible to the operator [31,32]. Constraints refer to the task- and goal-relevant information (eg, how far is the patient’s BP from optimal values? Are the patient’s hemodynamic parameters changing as expected?). In most cases, EDs are GDs in the sense that they typically also use shapes and colors to facilitate improved assimilation of the patient’s current state by the clinician, but a GD cannot always be classified as an ED.

**Figure 26:**
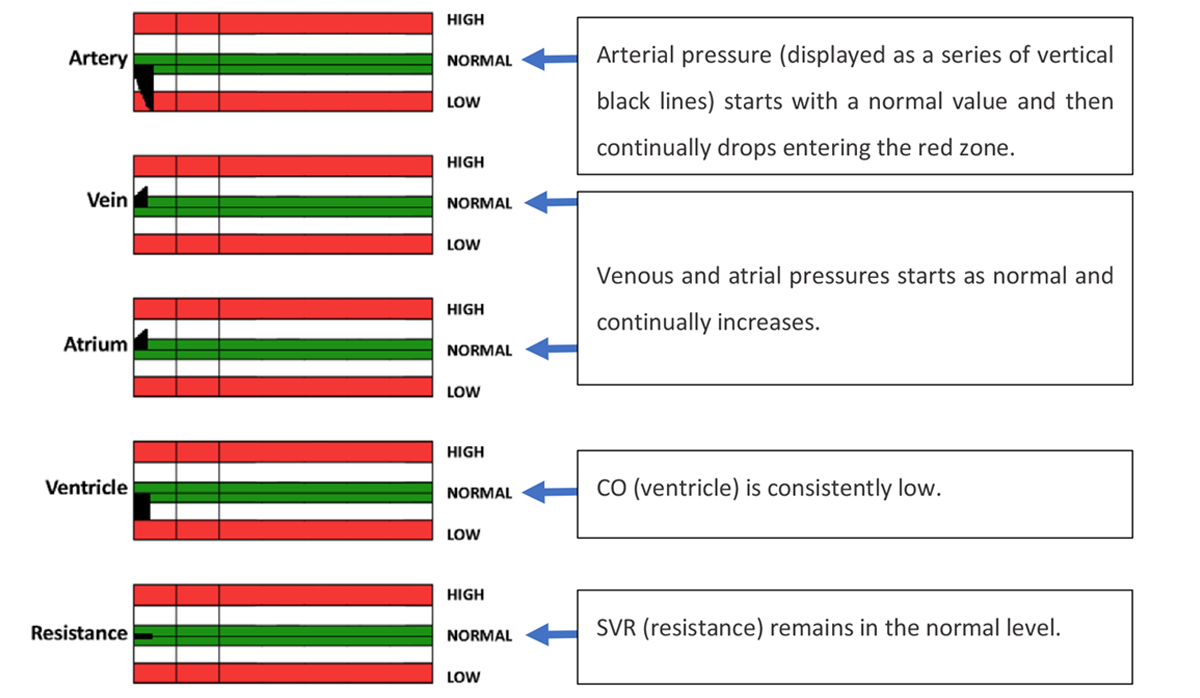
The strip-chart display displayed the 5 variables separately using 55 × 660-pixel bar graphs. Every second, the graphs were updated, and a new bar was added to the graph. In this scenario, the strip-chart display started with all variables in the normal condition and quickly evolved to a low heart strength state. This image is a model of the concept presented in this paper.

Effken et al [32] developed 2 EDs for hemodynamic data visualization, namely an integrated balloon display (IBD) and an etiological potential display (EPD). The 2 EDs were compared with a traditional strip chart display (TSD), which displayed the data using the single-sensor-single-indicator model and was considered by the authors the traditional display (Figure 26). The TSD displayed trends for the arterial, venous, and atrial pressures; CO; and SVR. The terms used for the variables in the 3 displays differed somewhat from the terms used in critical care. For example, *SVR* was replaced by *resistance* and *CO* was replaced by *ventricle*. The rationale for more generic physiological labels instead of the conventional ones was that the authors wanted to investigate the utility of the display by students with no clinical experience as well as by experienced participants.

**Figure 27:**
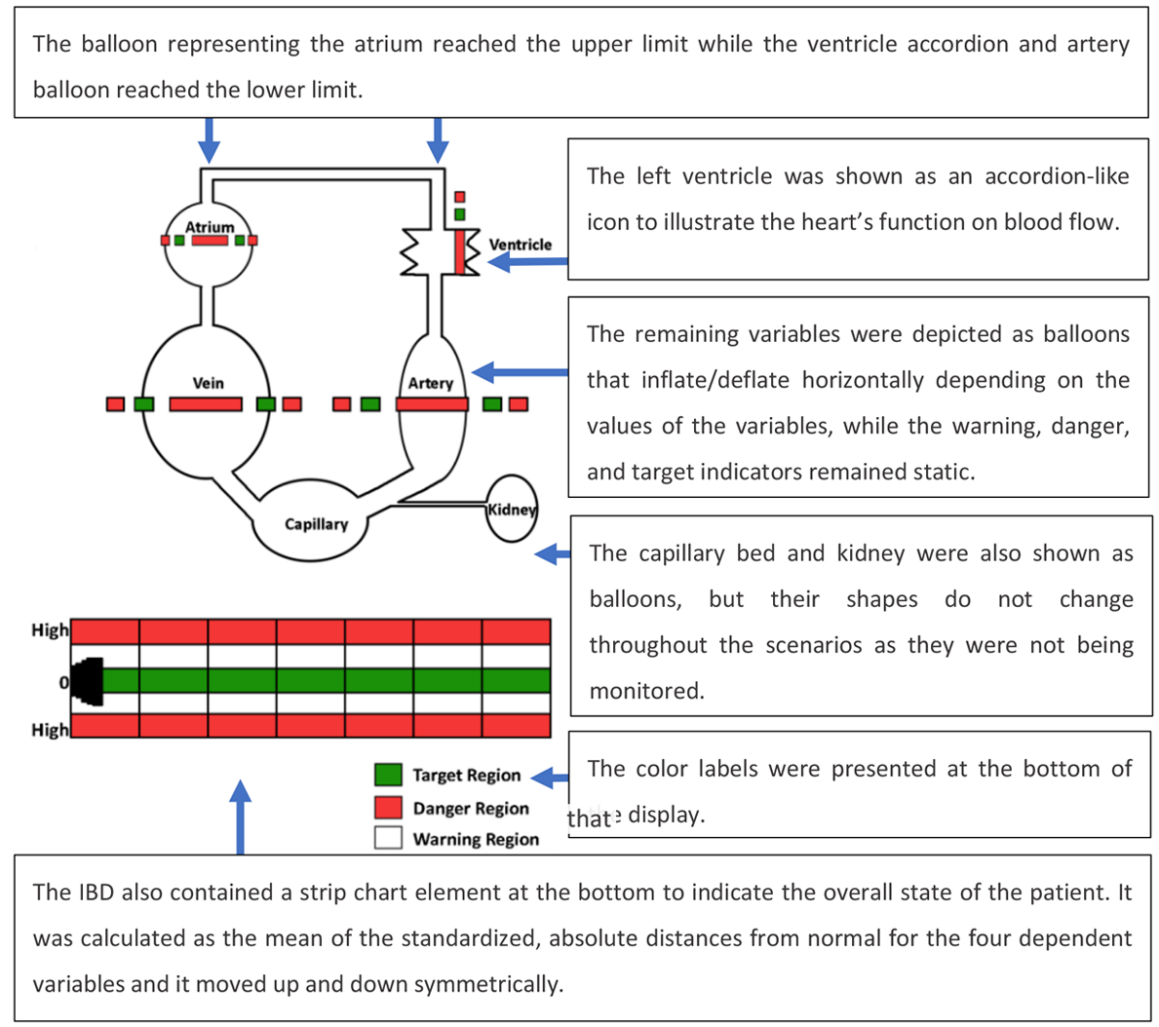
Hemodynamic variables presented using an integrated balloon display, where each system was presented in the form of balloons that can be expanded or shrieked according to the value of the variable. This image is a model of the concept presented in this paper.

**Figure 28:**
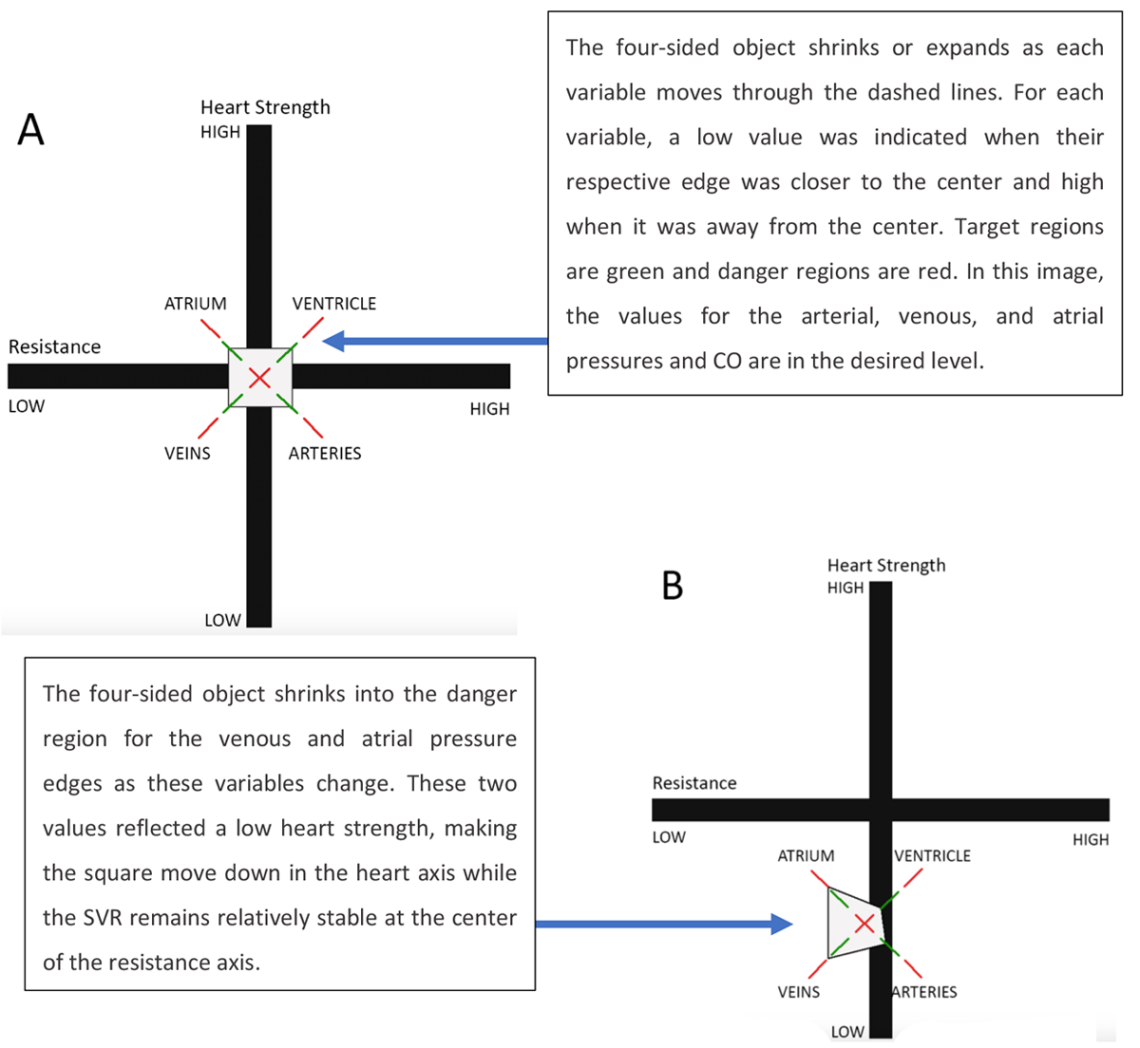
Hemodynamic data are presented using the etiological potential display in a normal state (A) and in an abnormal state (B). The vertical axis represented heart strength and the horizontal axis represented systemic vascular resistance. Fluid changes were shown as a shrinking or expanding square. The central crossing point for each bar (axis) represented the optimal value for each. This image is a model of the concept presented in this paper.

The IBD (Figure 27) represents each system in the form of balloons that expanded or shrunk according to the value of the variable. Colored regions around the balloons represent different states: good (green), warning (white), and danger (red). The IBD also contains a strip chart element at the bottom to indicate the overall status of the patient. In the EPD (Figure 28), the vertical axis represented heart strength and the horizontal axis represented resistance. Fluid changes were shown as a shrinking or expanding square. The central crossing point for each bar (axis) represented the optimal value for each. Figure 28 presents the patient data in a *normal* state (top left image) and in a low heart strength state (bottom right image), where the values of pressure and flow have moved away from the targeted state, deforming the 4-sided object and moving it away from the central crossing point of the resistance and heart strength axes.

An experiment was carried out with 6 experienced nurses and 6 student nurses. Participants were asked to treat a simulated patient using simulated drugs, based on a clinical assessment of the data presented on the monitor, to get their patients’ vital signs into the normal range as quickly as possible. It was observed that both groups of nurses initiated the treatment faster, used fewer drugs, and were able to maintain the vital signs within the target range for longer when using the ED in comparison with the TSD. In addition, the student nurses using the EDs were able to match the performance of experienced nurses using the traditional display.

The novel concepts presented by Effken et al [32] are quite innovative, and the study demonstrated the potential to enhance nurses’ performance in critical care. However, there were some issues with the experimental design that could have biased the results. For example, considering that the TSD does not resemble a typical PM, as presented in Figure 1, it is not clear that the TSD was a valid control display. In addition, while the experienced clinicians were instructed regarding the terminology changes so that they could relate the new terms to the ones actually used in clinical practice; however, it is unclear what impact these changes in the mental model had on the experienced clinicians. This may help explain why student nurses using the EDs were able to match the performance of more experienced clinicians.

**Figure 29:**
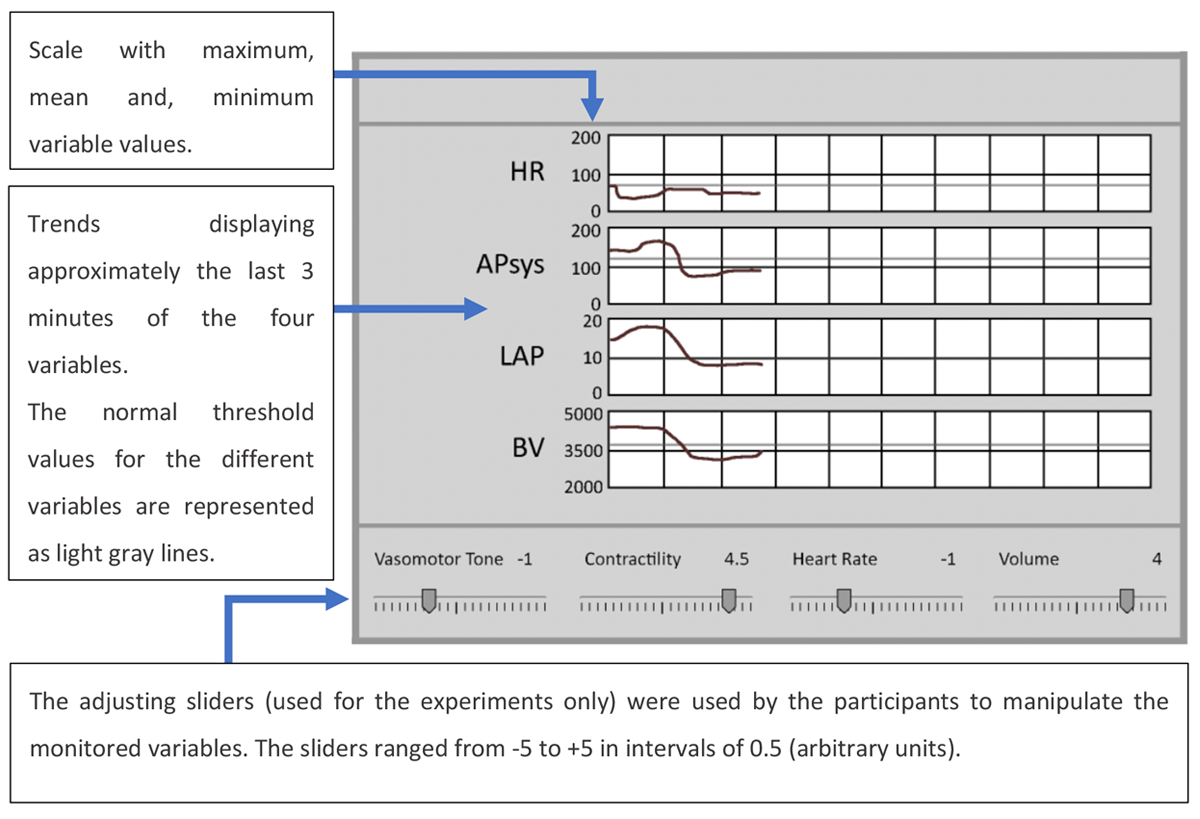
Trend display used by Jungk et al as a control display (a model of the concept presented in the paper). The trend display presented the heart rate (bpm), systolic arterial pressure (mmHg), left atrial pressure (mmHg), and blood volume (mL).

Jungk et al [33] developed a profilogram display and an ED and compared these 2 novel displays to a trend display. Similar to the main interface of the traditional PM, the trend display, presented data using the single-sensor-single-indicator approach. It is possible to configure most commercial PMs to present data using the trends format, but it was reported that this functionality of the PM was infrequently used in critical care [30]. The trend display was used to monitor HR, systolic arterial pressure (APsys), LAP, and blood volume (BV). As the data were presented using the trends format only, to know current values for each variable, the user had to interpolate the values visually with the aid of the trend display scales. The time axis range for each variable was between 0 and 10 min (Figure 29).

**Figure 30:**
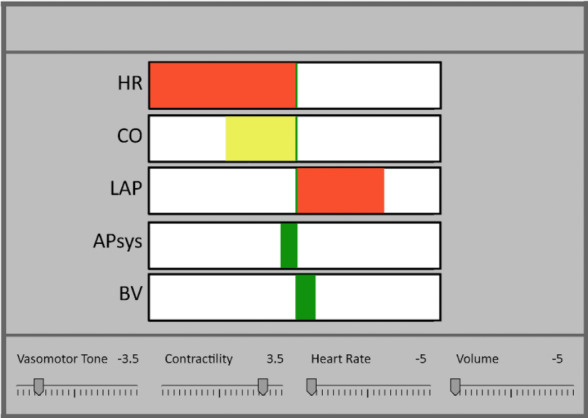
Profilogram display used by Jungk (a model of the concept presented in the paper). Profilograms for HR (too low), CO (a little low), LAP (too high), APsys and BV (good) were displayed.

As a part of the experiment, at the bottom of the 3 displays, the researchers added a control panel that was used to manipulate 4 functional parameters: HR, vasomotor tone, contractility, and circulating BV. The profilogram display was developed based on the principle of intelligent alarms. This system combined the relevant data needed by the physician to make decisions (eg, each monitored variable, physiological background knowledge, and patient-specific knowledge). The system used fuzzy logic to generate color-coded profilograms (Figure 30) [34]. Each profilogram presented the amount of a variable’s deviation in a positive or negative range for its related variable (HR, APsys, LAP, BV, and CO). Normal values for the variables were represented as a line in the middle of each profilogram. Bars to the left side of this line indicated a state variable becoming *too low* and bars to the right side of this green line indicated a state variable becoming *too high*. The amount of deviation was indicated by the length and the color of the bar (green for normal values, yellow for small deviations, and red for excessive deviations), which was intended to support rapid perception of the patient’s state.

**Figure 31:**
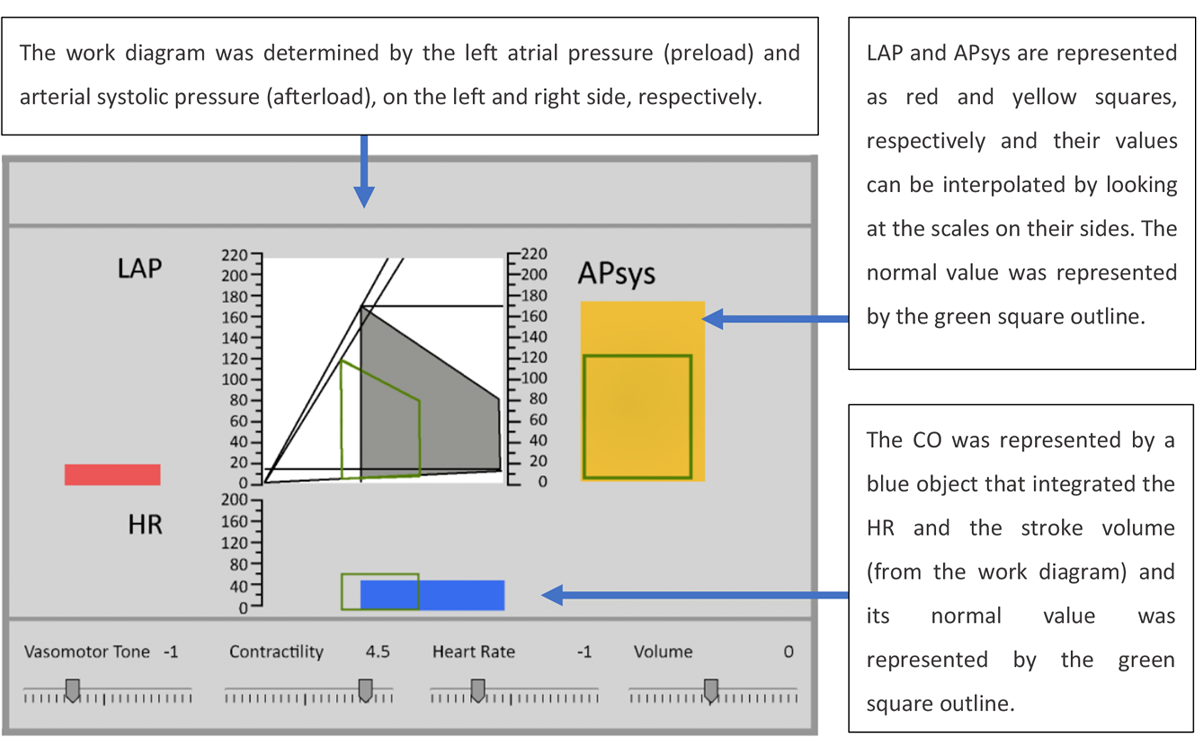
Jungk's (1999) ecological displays (a model of the concept presented in the paper).

The third display evaluated by Jungk et al [33] was a simplified ED for hemodynamic monitoring that integrated the necessary components for decision making (Figure 31). The LAP, APsys, and HR were displayed according to their physical location in the heart and corresponding to the schematic work diagram of the heart, which was displayed in the center of the display. Some of these variables were displayed using the graphical object concept typically used by GDs [15-18].

A total of 20 anesthesiologists, with no previous experience with an ED or profilogram display, carried out a prescribed task on the 3 displays separately. They were required to observe the data presented on the screen and maintain the vital signs within the desired range by adjusting the sliders located at the bottom of the interface. The sliders corresponded to vasomotor tone, contractility, HR, and volume.

It was observed that participants finished the task with the monitored variables within the acceptable range more often when using the ecological interface than when using the other 2 displays. However, the performance of the participants in terms of time to complete the task, number of slider interactions, and time to find relevant information was found to be much quicker with the trend display than when using the ED or profilogram display. On the basis of these results, the authors concluded that participants performed better with the trend display. Jungk et al [33] hypothesized that the difference in the performance of the 3 displays was attributed to the years of experience anesthesiologists had with the trends display and suggested that the future ED designs should not differ too much from the traditional PM displays.

**Figure 32:**
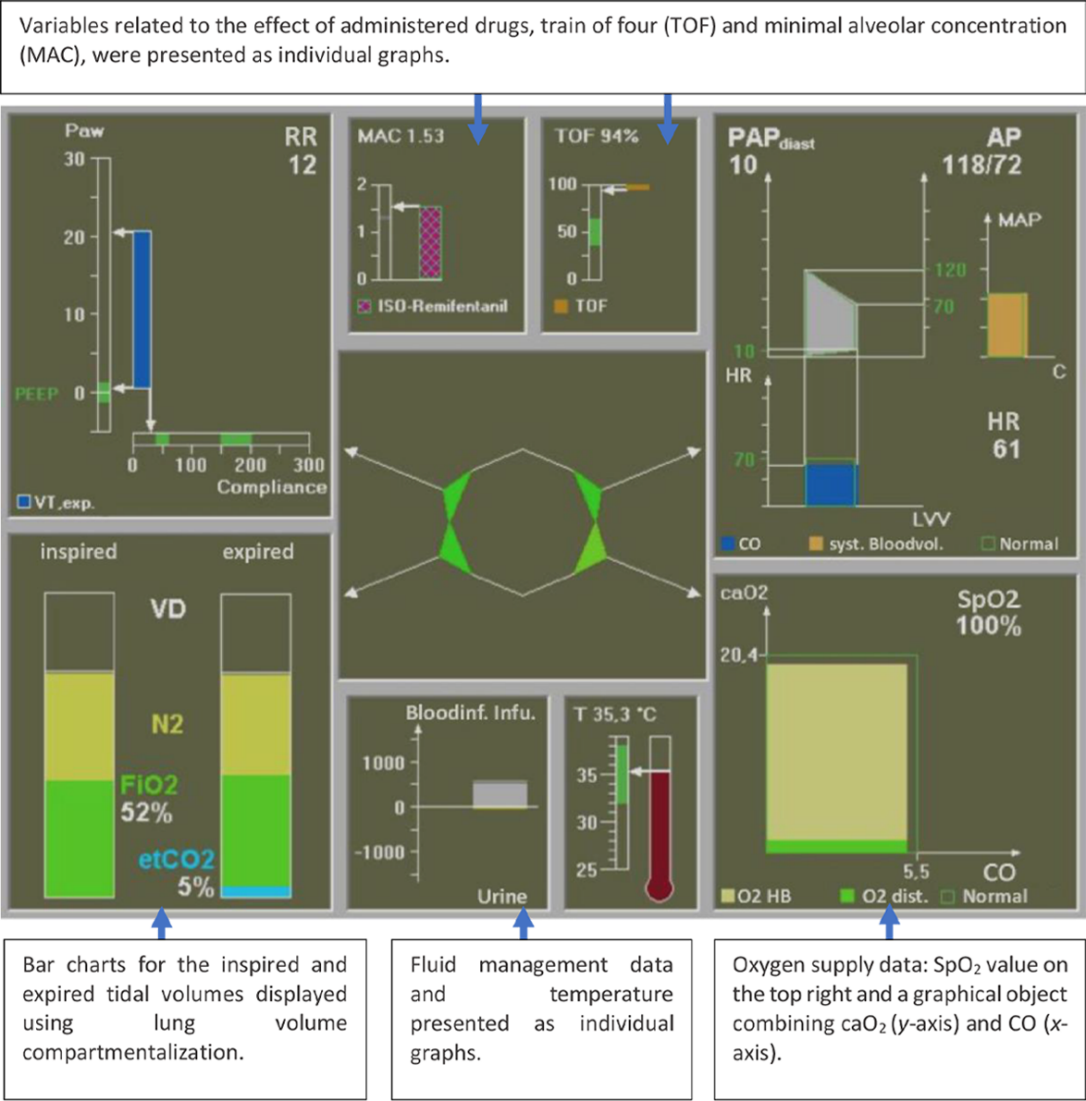
Jungk's ecological displays (first approach).

**Figure 33:**
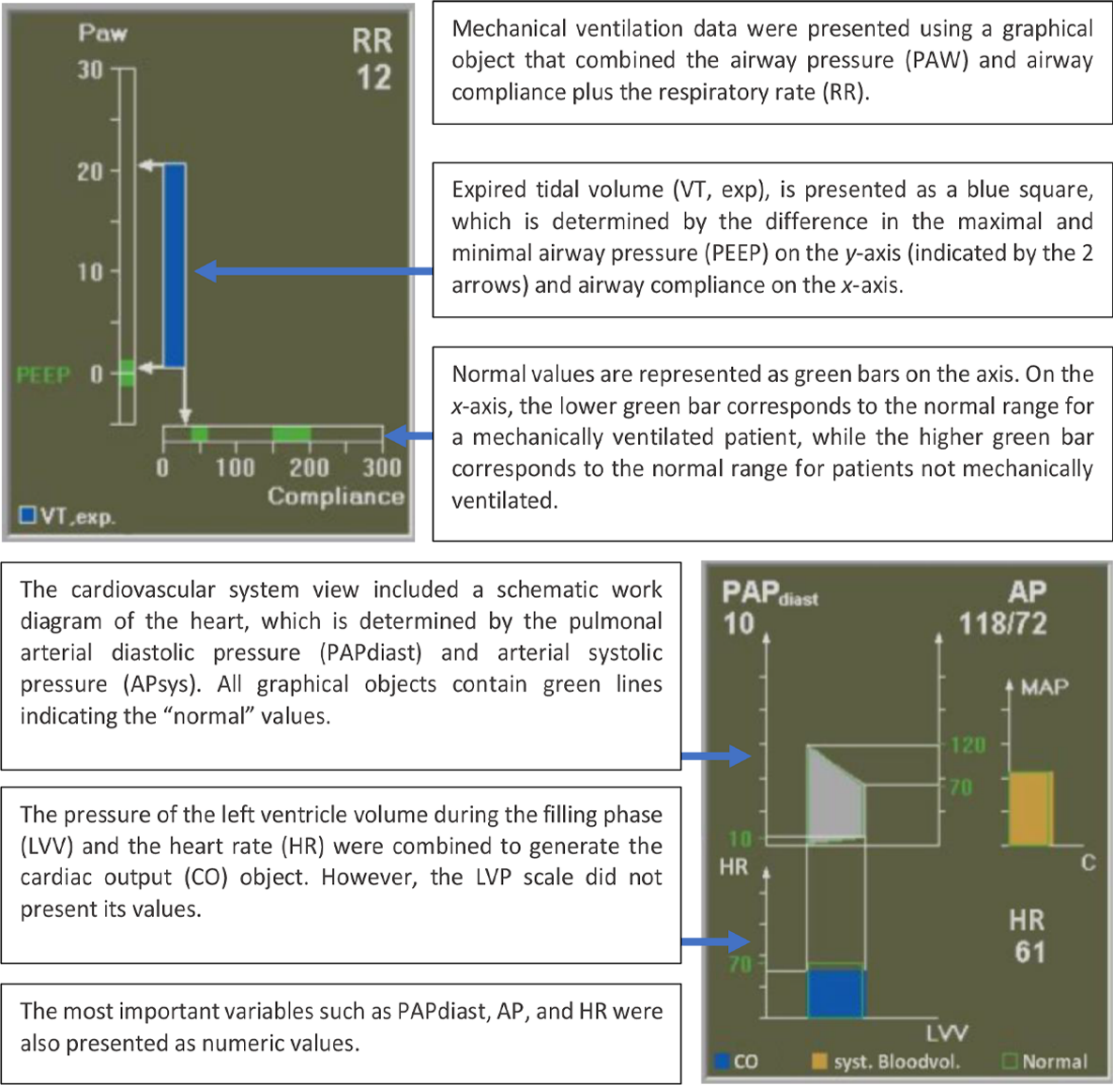
Respiratory and cardiovascular views used in experiment 1 on the Jungk et al (2000) study.

One year later, Jungk et al [35] developed an ED that presented 35 monitored variables, intending to support anesthesiologists during anesthesia monitoring. The reason for such a large number of monitored variables is that this ED (Figure 32) integrated data from different devices, such as a PM, a mechanical ventilator, and infusion pumps. This display made extensive use of graphical objects such as those presented in Figure 33.

The display was composed of 7 sections in which related variables were grouped, with a star in the middle, which represented an assessment of respiratory mechanics, respiratory volumes, oxygen supply, and the cardiovascular system. The star was color-coded based on the assessment of parameter constellations with the help of fuzzy sets and fuzzy rules. Jungk et al [35] intended to evaluate whether the performance of anesthesiologists would improve with the addition of an ED. The performance was assessed based on trial time, number of successful trials, and on some strategic behavior parameters (region-of-interest, related metrics, and think-aloud protocol). Of which, 16 anesthesiologists were asked to anesthetize a simulated patient under intervention conditions (the ED in conjunction with a simulated gas monitor and a simulated commercial PM) and control conditions (a simulated gas monitor and a simulated commercial PM only).

It was found that participants using the ED had poorer performance than the control group. For example, all participants correctly identified the blood loss scenario in the control group, while 3 participants failed in the intervention group. The eye-tracking analysis revealed that in the intervention group, almost half of the time, the ED was used as the main source of information and was frequently favored when identifying an evolving critical incident. It was also noticed that some of the elements in the ED, such as temperature and fluid management, were of little interest to the participants. Interestingly, 8 participants did not use the traditional PM when the ED was available.

**Figure 34:**
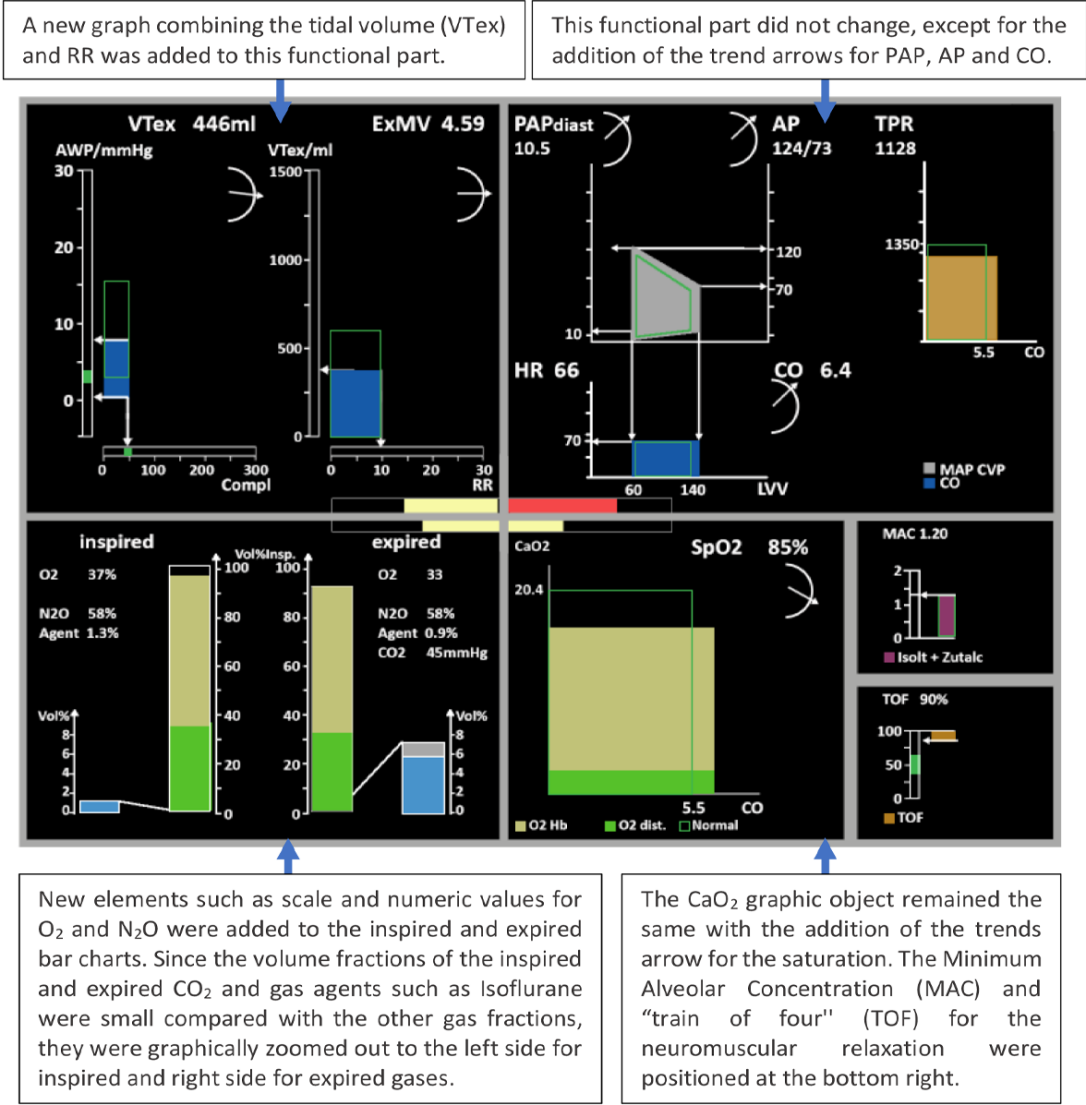
Jungk et al ecological displays (second approach). Profilogram bars based on the fuzzy logic approach for intelligent alarms were displayed at the center of the ecological displays, providing an overall state for each functional part of the display.

With the knowledge gained from this first experiment and following several interviews with anesthesiologists, Jungk et al [35] redesigned the ED to improve its usability (Figure 34). The data were rearranged on-screen to prioritize elements of most interest to the participants based on the eye-tracking analysis. In addition, this new display incorporated elements that had been used in other studies, such as the meters (gauge icons) and profilograms (Figure 34). Four color-coded profilograms were added to the center of the display representing groups of variables (respiratory mechanics, respiratory volumes, oxygen supply, and the cardiovascular system). The star in the middle was removed, as well as the temperature and fluid management variables, and the positions of the graphs were changed.

Jungk et al [35] repeated the same experiment with 8 different anesthesiologists using only an intervention group (no control). All participants identified the blood loss incident in this second test, but 1 participant did not identify the cuff leakage incident. The identification time was significantly shorter for both scenarios compared with the control test in experiment 1. This study exemplifies the importance of an iterative design process in which end users test the device in simulations.

**Figure 35:**
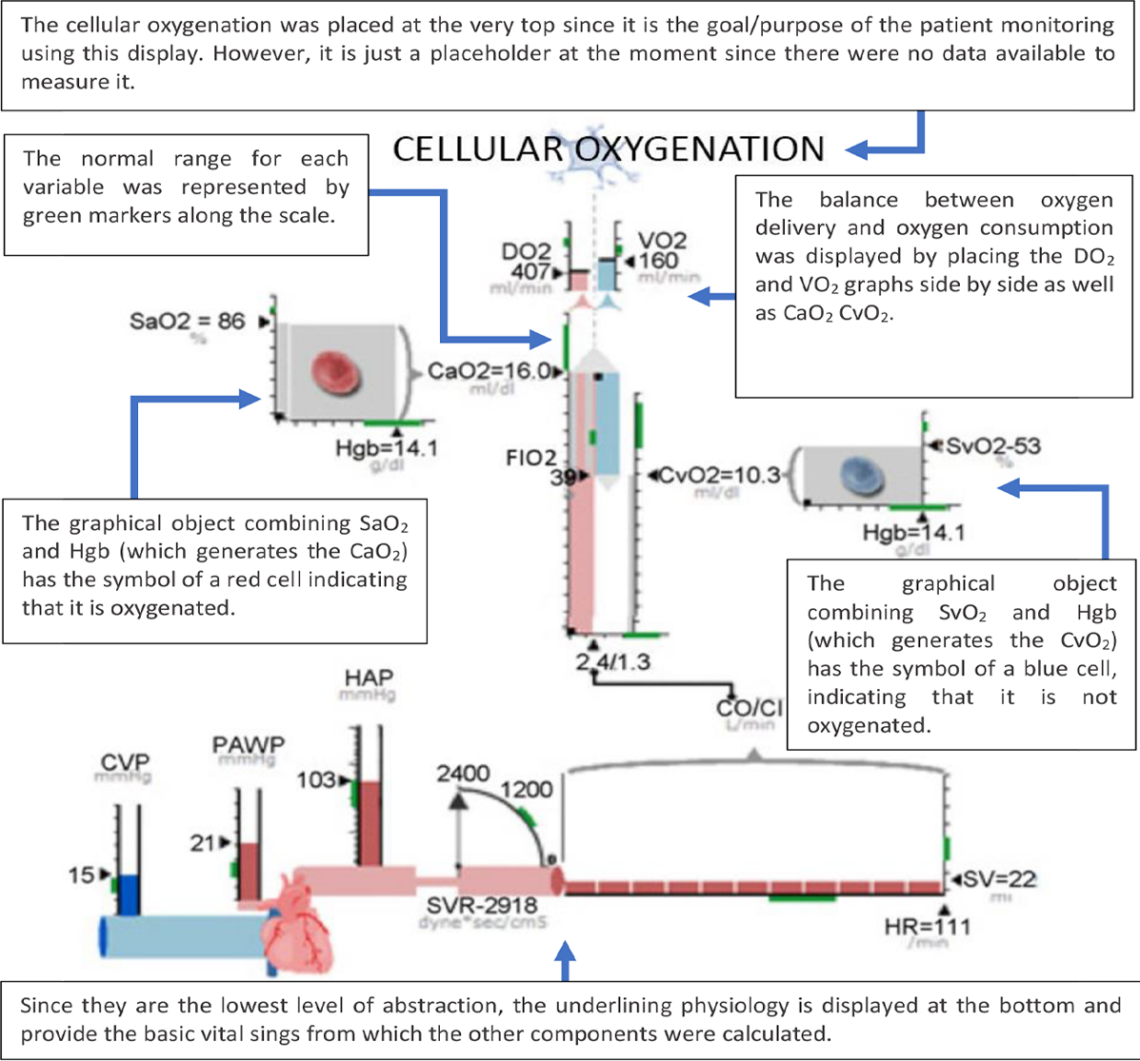
The Effken et al ecological display presented clinical data structured at 4 levels: purpose, balance, processes, and physiology.

A total of 11 years after the first experiment with an ED for patient monitoring, Effken et al [36] developed and evaluated an ED specifically designed for oxygen management. The development of the ED started with a cognitive work analysis (CWA) aimed to identify the work domain constraints and the cognitive tasks performed by ICU nurses. This helped the designers in arranging the elements on the screen to optimize the cognitive performance of the nurses. As a result, an interesting concept was developed. Figure 35 presents the clinical data structure at 4 levels: purpose, balance, processes, and physiology. The main goal of the system was cellular oxygenation, which was the *purpose*; therefore, it was placed on the top of the screen. If oxygenation was inadequate, the clinician then evaluated the *balance* between the variables related to oxygen demand and delivery, such as oxygen delivery (DO_2_), arterial blood oxygen content (CaO_2_), and oxygen consumption (VO_2_), which were presented in the form of bar charts directly below cellular oxygenation.

Depending on which side was out of balance, the clinician could identify the cause of the problem in either DO_2_ or metabolic *processes* (SaO_2_, Hgb, and CO), which were presented as graphical objects. Their underlying *physiology* (CVP, pulmonary artery wedge pressure, MAP, SVR, SV, and HR were presented as bar charts [37].

The ED was compared with a bar graph display (BGD) in terms of clinical event recognition, treatment efficiency, and usability. The BGD presented the monitored values as bar charts using the single-sensor-single-indicator model. In both displays (ED and BGD), the patient history was provided at the bottom of the display and the treatment options (clickable buttons) were presented on the right side of the display. In the experiment, 32 ICU nurses were asked to identify changes in the patient’s variables and use the available *treatments* to maintain these variables within the desired ranges.

The results showed no significant differences in the time to initiate the treatment between the ED and BGD. The mean percentage time in the target range varied for each display depending on the number of variables being presented simultaneously and the order of the experiment. Perceived workload (measured by the NASA-TLX questionnaire) was not statistically significantly different across displays.

As in the previous experiment by Effken et al (Effken et al, 1997) [32] there was no indication that the control display (BGD) was clinically used, which makes it impossible to draw meaningful comparisons between the novel display and the conventional PM.

## Discussion

### Principal Findings

This review aimed to critically review and examine the innovations in PM design proposed by researchers and to explore how clinicians responded to these novel design approaches. These proposed innovations are fully described in the Results section of this review. Having analyzed the methodologies used to develop and test these displays, as well as the results of these tests, a few topics have emerged for discussion.

Most novel displays described in this review were developed to promote rapid detection and interpretation of changes in patient vital signs, provide a *bigger picture* of the patient state, and reduce the physical and cognitive load of users and increase the SA for nurses and doctors. For example, GDs and object displays were developed by utilizing shapes and colors to represent changing vital signs. It was expected that these displays would better support nurses and doctors by reducing their detection and decision times and by improving diagnostic accuracy. However, in most cases, the performance of the participants when using the novel displays varied according to the test scenario. Statistically significant improvements in performance metrics were found when using a GD over a traditional PM for some scenarios, but not all of them [18-20,28,33,36]. Only three studies that evaluated a GD observed significant improvement for all tested scenarios [15,17,32], although it is important to mention that in these cases, a conventional PM was not used as a control. For example, one of these studies used as a control display not commonly used in real practice [32] while the other two used alpha-numeric displays as a control, which only presented the numeric values of vital signs without waveforms [15,17]. A traditional PM display in critical care will typically be composed of numeric values and waveforms. Therefore, it is not possible to determine if the outcomes would be the same if a traditional PM was used as a control in these cases.

In the studies where a novel PM was developed with the intention to improve the performance of clinicians by integrating information from several devices into a single screen, participants performed better when the volume of information presented simultaneously on-screen was not overwhelming [24,25] and when the *look and feel* of the traditional PM was not radically changed to accommodate the data integration [27]. When the number of variables presented on a single screen was excessive (eg, more than 30 variables), the cognitive load created for the user was too high, and the designers decided to make use of graphical and object elements to facilitate the assimilation of the patient’s state by the clinician [16,35]. Once again, it was verified that statistically significant improvements in the users' performance were found in some scenarios, but not for all scenarios. Therefore, when integrating data from multiple devices, it is important to display only those variables that are essential for the task at hand. This saves the user from feeling overwhelmed by the volume of information presented. Furthermore, challenges of data integration from multiple devices onto one screen go beyond usability and data visualization challenges, as medical devices might not always provide the technological means of integration.

### User Involvement in the Design Process

Before 2010, most studies did not mention end-user involvement during the design process. Some of these studies based the design of their interfaces on frameworks, such as EID [32,35,36] and CWA [17] or did not describe the design process used at all [16,19,33]. Other studies did not develop the interface from scratch, instead they tested previously developed displays [15,18] or presented adaptations of existing displays [20,35]. The majority of these studies had inconclusive results when they compared the performance and user satisfaction between the experimental and the conventional displays. On the other hand, generally, studies that used user-centered design (UCD) or participatory design approaches [24,25,27,28] had more satisfactory results regarding usability. One compelling case of how the interface design benefitted from user involvement in the design process can be seen in Jungk et al [35]. The authors conducted an initial study with an experimental display designed based on EID [17]. The results of this first attempt were not satisfactory, and the display was adjusted based on the results of the first experiment and several interviews with the end users. After making adjustments to the design following this feedback, the second experiment had superior results compared with the first experiment. It is worth noting that although the nurses and doctors are the end users of the PM and that design changes in this device will directly influence their user experience, the patient is the one who will ultimately benefit or be affected by the design of the PM.

### Study Design Considerations When Testing a Novel Patient Monitor

An essential usability attribute that is not given proper attention in the reviewed studies is safety, and the authors of the studies reviewed did not make references to how they addressed error prevention or error recovery in their displays. As seen with the polygonal display by Gurushanthaiah et al [15], it is possible for a novel display to be seen to enhance a clinician’s performance and to elicit a positive user experience, while also being likely to result in inadvertent use errors due to design limitations. Therefore, it is imperative that testing of novel displays also targets the identification of sources of use errors in the design. As a result, it is highly recommended that researchers conduct usability inspections on novel devices before user testing. One way to achieve this is through a heuristic analysis of the display in which clinical or human factors experts evaluate the device or system by assessing how it conforms to well-established user-interface design rules or heuristic guidelines, such as the usability heuristics proposed by Jackob Nielsen [5,38]. A review using the heuristics by Neilsen will not only highlight safety issues but will also identify if usability best practice is adopted in the display design around issues such as the visibility of system status, user control and freedom etc. None of the studies reviewed made reference to carrying out a heuristic analysis.

It should be made clear in a study design if the novel display is intended to replace or to augment a traditional PM. This consideration will heavily influence the introduction of a novel PM in a clinical context. For instance, clinicians might be willing to introduce a novel PM in their workflow as long as conventional equipment is not being removed. In cases where the novel PM is designed to fully replace a traditional PM and, if the novel PM’s interface differs significantly from that of a traditional PM, a more effective approach could be having the novel PM augment the traditional PM and not replace it. Once it is confirmed whether or not the users have fully adapted to the novel PM, further actions can be decided.

Devices are designed to be used in specific contexts of use; therefore, when evaluating a novel PM, researchers should design experiments in which the user interacts with the device in a setting and under circumstances similar to those expected in the intended context of use. However, most of the novel PMs described in this review were tested in a context of use that did not match the expected real-world conditions (eg, laboratories and work offices instead of quasi-clinical settings). The outcomes of an experiment will be weakened if the experiment fails to replicate the expected context of use.

In addition, the control devices used during the testing should be as close as possible to the devices typically used by the users for this application. Some experiments have used an unrepresentative control display as a control for the novel PM [17,19,28,32,35,36]. In such cases, it is impossible to draw conclusions on how the novel PM may impact patient care in comparison with the current standard of clinical care and use.

If at all possible, researchers should provide a comprehensive program of training on the novel interface to participants before carrying out testing. The purpose here is to achieve as a high level of familiarization with the novel display, before testing, as is feasible. Essentially, one should try to eliminate lack of familiarity with the display as a *confounding factor* in the testing, as it is expected that the control display (typically the PM in regular use) will be very familiar to the participants.

This training should ideally include not only an introduction to the new display but also feature demonstrations, simulations, and competency tests.

Providing robust training on a new interface as part of a research study requires a considerable amount of effort and time and, in many cases, this can be very challenging. Nearly all studies reviewed did not exceed 45 min of training. Researchers must keep in mind that although a short training session may be sufficient to allow the participant to understand how the device works, it may not be enough to achieve the same level of familiarity as exists with the control device. In these circumstances, when a novel interface is compared with the standard approach, the standard approach likely achieves much higher preference and, therefore, distorted preference data can result.

Some studies evaluated novel PMs using research participants with no (or very little) medical background and the results of these studies were not presented in this review. The reason for this is that, although it is possible to introduce nonmedical participants to a display to be tested, participants who are not the intended users of a device will have completely different perceptions of the device and will likely use different cognitive strategies to interact with it. These differences produce inaccurate outcomes, as demonstrated by Gurushanthaiah et al [15]. Therefore, we recommend that only samples of the intended users of a device should be used as test participants.

Usability is defined by the ISO 9241-210 (section 2.13) as “the extent to which a system, product or service can be used by specified users to achieve specified goals with effectiveness, efficiency and satisfaction in a specified context of use.” Therefore, for good usability, a device must not only improve effectiveness and efficiency (eg, detection/response/trial times, treatment efficiency, accuracy, etc.) but also provide a positive experience for the user. Up to the early 2000s, most studies solely focused on performance metrics and neglected the effects of the design on the user’s experience, such as cognitive workload, comfort, and preference. However, since 2003, almost all studies have evaluated the effects of the design on the user during their experiments using questionnaires. For example, studies used either the NASA-TLX questionnaire to measure self-reported perceived workload [19-21,28] or Likert scales to measure participants' preference or satisfaction [22] or both [10,11,36]. The addition of such questionnaires as a part of the experimental methodology indicates a positive paradigm shift in which positive user experience and device satisfaction are also perceived as essential qualities to be considered in the design of a novel PM.

On the basis of our experience with reviewing these studies, we would propose the following recommendations for researchers designing and evaluating new PM interface designs:

1. To identify any usability problems associated with the design of user interfaces and to mitigate error risks before user testing, researchers should consider conducting a heuristic analysis of the displays.
2. During the user testing, the purpose of the novel PM should be made clear to the participants, including specifying whether the purpose of the novel PM is to augment or replace a conventional PM or not. This is important because this information will have an impact on users’ perceptions of the device during testing.
3. In all development stages of a novel PM, targeted end users (eg, ICU nurses and anesthesiologists) must be involved in the design and evaluation processes through a UCD methodology.
4. Researchers should strive to design a test protocol that accurately reflects the expected context of the use of the display.
5. To achieve meaningful results and a fair comparison, when testing a novel PM against a conventional PM, the control device (representing a conventional PM) must match the characteristics of the conventional PM as closely as possible.
6. Attempt to eliminate the participant’s lack of familiarity with the novel display (relative to their familiarity with the conventional PM) as a confounding factor in testing. Before testing a novel PM with potential end users, researchers should provide extensive training to the participants on the novel PM (preferably involving multiple training sessions) to acclimatize the participants to the use of the novel display and ideally achieve a high level of familiarity with it.
7. As user satisfaction is a key component of usability, more comprehensive assessments of user satisfaction should be carried out using both quantitative and qualitative analyses.

Although it is understandable that fulfilling some of these recommendations in a research context can be challenging because of resource and time constraints, by following them we believe that researchers can significantly enhance the quality of their research.

## Appendix

Multimedia Appendix 1 Summary of studies reviewed.

Multimedia Appendix 2 Quality assessment summary.

## Abbreviations

APsys: systolic arterial pressure
Art: arterial blood pressure
BGD: bar graph display
BP: blood pressure
BV: blood volume
CO: cardiac output
CSO: current state object
CVP: central venous pressure
CWA: cognitive work analysis
DO_2_: oxygen delivery
ED: ecological displays
EID: ecological interface design
EPD: etiological potential display
GD: graphical display
HR: heart rate
IBD: integrated balloon display
ICU: intensive care unit
IGD: integrated graphical display
LAP: left atrial pressure
MAP: mean arterial blood pressure
MV: minute volume
NIBP: noninvasive blood pressure
OR: operating room
O_2_: percentage of inspired oxygen
PAC: pulmonary artery catheter
PAD: diastolic pulmonary artery pressure
PEEP: positive end–expiratory pressure
PM: patient monitor
PVR: pulmonary vascular resistance
SA: situation awareness
SaO_2_: arterial blood oxygen saturation
SpO_2_: blood oxygen saturation
SV: stroke volume
SVR: systemic vascular resistance
TSD: traditional strip-chart display
UCD: user-centered design
