# Supplementary material for: Novel Interface Designs for Patient Monitoring Applications in Critical Care Medicine: Human Factors Review
Source: JMIR Hum Factors. 2020 Jul 3;7(3):e15052. doi: 10.2196/15052 (PMC7367533; doi:10.2196/15052)
Supplement: Multimedia Appendix 1 [file humanfactors_v7i3e15052_app1.docx]

# Appendix 1 – Summary of Studies Reviewed

The 16 studies that matched the inclusion criteria regarding trials with novel displays are summarized in the table below.

| **Source** | **Control display** | **Intervention display** | **Context of Use** | **Intended user (subjects)** | **Development**  **Method** | **Training** | **Scenarios** | **Metrics** |
| --- | --- | --- | --- | --- | --- | --- | --- | --- |
| Gurushanthaiah et al  (1995) [15] | Numeric display without waveforms | Graphical display | Operating Room | 13 anaesthesia residents  (within-subjects) | N/A | Introduction to the displays followed by 2 minutes of scripted training | No specific scenario. Changes in the variable values | Response time ▼  Accuracy ▲ |
| Effken et al (1997) [32] | Strip Chart  (not clinically used) | Ecological display | ICU | 6 ICU nurses and 6 student nurses  (within-subjects) | Based on the principles of EID | 20 to 30-min training session followed by a practising session until participants became comfortable with the use of the display | Hypertension, heart failure and hypovolemia | Time to initiate  treatment ▼  Number of drugs used ▼  Treatment efficiency ▲ |
| Michels et al (1997) [16] | Traditional PM  + other monitoring devices | Integrated graphical display | Operating Room | 10 faculty member anaesthesiologists  (between-subjects) | Not informed | 15-min introduction to the new display | Blood loss, inadequate paralysis, cuff leak and depletion of soda lime | Detection time ▼  Identification time ▼ |
| Blike et al (1999) ^16^[17] | Numeric display without waveforms | Graphical display | Operating Room | 11 anaesthesiologists  (within-subjects) | Cognitive analysis based on user interviews | Pre-test questionnaire followed by multiple trials with both displays | Cardiac shock | Response time ▼  Accuracy ▲ |
| Jungk et al (1999) [33] | Trend display  (not clinically used) | Profilogram and  ecological displays | Operating Room | 20 anaesthesiologists  (within-subjects) | Not informed | 20-30 min to familiarize with the task, displays and control actions | No specific scenario. Changes in the variable values | Time to complete the task  Slider interactions  Treatment efficiency ▲ |
| Jungk et al (2000) [35] | Traditional PM  + other monitoring devices | Integrated graphical and  ecological displays  +Traditional PM | Operating Room | 16 anaesthesiologists  (within-subjects) | Based on the principles of EID and previous display (Jungk et al, 1999) | 45 min to familiarize with the display | Blood loss and cuff leakage | Identification time  Event identification time |
|  | Traditional PM  + other monitoring devices | Integrated graphical and  Ecological displays  +Traditional PM | Operating Room | 8 anaesthesiologists | Based on the analysis of the results of study 1 | 45 min to familiarize with the display | Blood loss and cuff leakage | Identification time ▼  Event identification  time ▼ |
| Y. Zhang et al (2002) [18] | Commercial PM | Graphical display | Operating Room | 12 anaesthesiologists (residents and faculty members)  (within-subjects) | Same display developed by Blike et al (1999) | Introduction to the new display followed by training (including competency test) | Hypovolaemia, arrhythmia, ischaemia and bronchospasm | Detection time ▼  Event recognition time  Situation awareness ▲ |
| Agutter et al (2003) [19] | Numeric display without waveforms +  Commercial PM | Graphical display + Commercial PM | Operating Room | 20 anaesthesiologists  (between-subjects) | Not informed | Pre-test questionnaire followed by a 15-min training session | Anaphylaxis during a total hip replacement and blood loss and myocardial ischemia during a radical prostatectomy | Detection time ▼  Diagnosis time  Treatment time ▼  Self-reported workload |
| Wachter et al (2006) [21] | Numeric display without waveforms +  Commercial PM + Anaesthesia Machine | Graphical display + Commercial PM + Anaesthesia Machine | Operating Room | 19 anaesthesiologists (residents and faculty members) | Iterative design process | 15-min training session | Obstructed endotracheal tube, endobronchial intubation, intrinsic PEEP, hypoventilation and stable scenario | Detection time ▼  Treatment time ▼  Accuracy ▲  Self-reported workload ▼ |
| Albert et al (2007) [20] | Commercial PM | Graphical display + Commercial PM | Operating Room | 16 anaesthesiologists (residents and faculty members)  (between-subjects) | Display developed by Agutter et al (2003) adjusted | Pre-test questionnaire followed by 10-min scripted overview of the simulation equipment and 15-min training video about the IGD | Hypertension, myocardial ischemia, haemorrhagic hypovolemia, left ventricular failure, septic shock and acute respiratory distress syndrome + myocardial ischemia). | Treatment performance ▲  Detection time ▼  Treatment time ▼  Self-reported workload |
| Effken et al (2008) [36] | Bar graph  (not clinically used) | Graphical and  ecological displays | ICU | 32 ICU nurses  (within-subjects) | Based on the principles of EID and cognitive work analysis | Pre-test questionnaire followed by training with each display | 4 scenarios with variations on the patient history (age, disease, vital signs etc.) | Event recognition  Treatment efficiency ▲ |
| Tappan et al (2009) [22] | Traditional PM | Traditional PM with visual cues | Operating Room | 22 anaesthesiologists (residents and faculty members)  (within-subjects) | Not informed | Pre-test questionnaire followed by an introduction to the new display | Light anaesthesia, pulmonary embolism, anaesthetic overdose, hypovolemia, malignant hyperthermia and blood pressure fluctuation | Detection time ▼  Missed events ▼  Satisfaction |
| Görges et al (2011) [10] | Commercial PM  + infusion pump | Integrated graphical display | ICU | 16 ICU nurses  (within-subjects) | User-centred design | 3.5 to 7-min training followed by a competency test | 6 scenarios with variations on the parameter values | Decision time ▼  Accuracy ▲  Self-reported workload  Display preference |
| Görges et al (2012) [11] | Commercial PM  + infusion pump | Integrated graphical display | ICU | 15 ICU physicians  (within-subjects) | User-centred design | 3.5 to 7-min training followed by a competency test | 6 scenarios with variations on the parameter values | Decision time ▼  Accuracy ▲  Self-reported workload  Display preference ▲ |
| Koch et al (2013) [27] | Traditional PM  + other monitoring devices | Integrated display | ICU | 12 ICU nurses  (within-subjects) | Observations+ user centred design | Self-paced training session using PowerPoint followed by a competency test | 3 scenarios to cover tasks related to medication management, patient awareness and team communication | Situation awareness ▲  Task completion ▲ |
| Drews and Doig (2013) [28] | Numeric display without waveforms | Graphical object display | ICU | 42 ICU nurses  (between-subjects) | Interviews + literature review + iterative design | 20-min training | Early sepsis, septic shock, pulmonary embolus and a stable scenario | Response time ▼  Accuracy ▲  Self-reported workload▼ |
| The arrow pointing up (▲) and down (▼) indicate that the metric tagged was superior when the intervention display was used in comparison to the traditional PM in at least one scenario. The ▲ symbol indicates increased values while the ▼ symbol indicates reduced values. | | | | | | | | |
