# Supplementary material for: Novel Interface Designs for Patient Monitoring Applications in Critical Care Medicine: Human Factors Review
Source: JMIR Hum Factors. 2020 Jul 3;7(3):e15052. doi: 10.2196/15052 (PMC7367533; doi:10.2196/15052)
Supplement: Multimedia Appendix 2 [file humanfactors_v7i3e15052_app2.docx]

# Appendix 2 – Quality Assessment Summary

The items below were extracted and adapted from the Cochrane Collaboration’s tool for RCTs [11] and were used to assess the quality of the papers reviewed. Each item can be marked as “Low risk”, “High risk”, or "Unclear risk" depending on the information provided about the study design.

1. **Random sequence generation**: Risk of selection bias due to inadequate use or lack of a random component when distributing participants to control and intervention groups.
2. **Blinding of outcome assessment**: Risk of detection bias due to the knowledge of the allocated PM used (control or intervention) by the assessors.
3. **Incomplete outcome data**: Risk of bias due to attrition (participant drop-out) or exclusions of participants outcome data from the analysis.
4. **Selective reporting**: Risk of reporting bias due to the selection of a subset of the original variables recorded, based on the results, for inclusion in the publication.

The Cochrane Collaboration’s tool had two additional items “Allocation concealment” and “Blinding of participants and personnel” which were not included in our assessment since it is not possible to blind participants and personnel to the PM (control and intervention) used in the study. The Cochrane collaboration tool also suggests questions specifically for cross-over trials, which were also included in the analysis of the studies:

1. Was the use of a cross-over design appropriate?
2. Is it clear that the order of receiving treatments was randomised?
3. Can it be assumed that the trial was not biased from carry-over effects?

The quality assessment of the 16 studies that matched the inclusion criteria regarding trials with novel displays are summarized in the table below.

| **Source** | **Items for RCTs** | | | | **Questions for cross-over trials** | | | **Comments** |  |
| --- | --- | --- | --- | --- | --- | --- | --- | --- | --- |
|  | **1** | **2** | **3** | **4** | **1** | **2** | **3** |  |  |
| Gurushanthaiah et al  (1995) [15]  **Study 1** | N/A | Unclear | Low | Low | Yes | Yes | Unclear | Training and competency levels verification to determine adequacy. Sleep patterns, caffeine and alcohol intake and medication were taken into account before each experiment. |  |
| Gurushanthaiah et al  (1995) [15]  **Study 2** | N/A | Unclear | Unclear | Low | Yes | Yes | Unclear | 5 participants from experiment 1 were randomly chosen for the second experiment. Results from these participants were not detailed in the paper. |  |
| Effken et al (1997) [32]  **Study 3** | N/A | Unclear | Unclear | Low | Yes | Unclear | Unclear | Participants in studies 1 and 2 were not end-users (psychology undergraduates). The presentation order of scenarios was randomized. The computer recorded the amount and strength of each drug used and the number of keypresses made, but it is unclear if that dismisses the need for Blinding of outcome |  |
| Michels et al (1997) [16] | Low | Unclear | Low | Low | N/A | N/A | N/A | Participants in the control group had to page through four screen displays to observe the full range of clinical information, which does not reflect the real-world conditions |  |
| Blike et al (1999) [17] | N/A | Unclear | Low | Unclear | Yes | No | No | No randomization in the order of the displays during the testing. The control display was always the first. Pre-specified outcomes were reported, except data regarding the response time was not provided, only the difference in response time between the two displays. |  |
| Jungk et al (1999) [33] | N/A | Unclear | Low | Low | Yes | No | No | No information was provided regarding randomization order or displays. The variables started with a randomized value which makes it impossible to make sure each task had the same difficulty. Control task not clinically relevant. |  |
| Jungk et al (2000) [35]  **Study 1** | N/A | Unclear | Low | Low | Yes | No | No | No information was provided regarding randomization order or displays. The computer recorded the amount and strength of each drug used and the number of slider interaction, but it is unclear if that dismisses the need for Blinding of outcome. Three subjects had participated in our previous experiments and, hence, had some experience with ecological interfaces. |  |
| Jungk et al (2000) [35]  **Study 2** | N/A | Unclear | Low | Low | Yes | No | No | Experiment design similar to study 1. Very Small number of participants (8 anaesthesiologists). These were different from the ones in the first experiment. |  |
| Y. Zhang et al (2002) [18]  **Study 1** | N/A | Unclear | Low | High | Yes | Unclear | Unclear | It is not clear that the order of receiving treatments was randomised; therefore it is not possible to confirm if the results were not affected by the carry-over effect. Scenarios presented in random order. |  |
| Y. Zhang et al (2002) [18]  **Study 2** | N/A | Unclear | Low | Low | Yes | Unclear | Unclear | Participants were twelve first-year undergraduate students and not anaesthesiologists as in experiment 1. |  |
| Agutter et al (2003) [19] | Low | Unclear | Low | Unclear | N/A | N/A | N/A | Reviewers were not blinded to the display used, but the authors argued that data scoring was based on rigorous a priori criteria. All performance results were reported, but the numbers for the workload analysis were not reported. |  |
| Wachter et al (2006) [21] | Low | Low | Low | Low | N/A | N/A | N/A | Expert anesthesiologists assessing the results were blinded to the intervention method and a third assessor reviewed discrepancies between the reviewing anesthesiologists. | |
| Albert et al (2007) [20] | Low | Low | Low | Unclear | N/A | N/A | N/A | Stratified Randomisation used to balance by expertise (anaesthesiologists and anaesthesia residents), but no information was provided regarding the randomization algorithm. Assessors were blinded to the intervention method. |  |
| Effken et al (2008) [36] | N/A | Unclear | Low | Low | Yes | Yes | Yes | Two nurses did not view all scenarios; therefore, they were not included in the study. The randomisation algorithm was not explained. Results show a high carry-over effect. |  |
| Tappan et al (2009) [22] | N/A | Unclear | Low | Unclear | Yes | Yes | Unclear | The order that subjects used the displays and the order of the scenarios within each experiment period was decided randomly using computer-generated balanced permutations. |  |
| Görges et al (2011) [10] | N/A | Unclear | Low | Low | Yes | Yes | Unclear | Randomization algorithm was not described. |  |
| Görges et al (2012) [11] | N/A | Unclear | Low | Low | Yes | Yes | Unclear | Randomization algorithm was not described. |  |
| Koch et al (2013) [27] | N/A | Unclear | Low | Low | Yes | Yes | Unclear | Training using a PowerPoint presentation (20-30 minutes). |  |
| Drews and Doig (2013) [28] | Low | Unclear | Low | Low | N/A | N/A | N/A | No information was provided regarding the randomization algorithm. |  |
